# Spatial analysis and CD25-expression identify regulatory T cells as predictors of a poor prognosis in colorectal cancer

**DOI:** 10.1038/s41379-022-01086-8

**Published:** 2022-04-28

**Authors:** Christian H. Bergsland, Marine Jeanmougin, Seyed H. Moosavi, Aud Svindland, Jarle Bruun, Arild Nesbakken, Anita Sveen, Ragnhild A. Lothe

**Affiliations:** 1 Department of Molecular Oncology, Institute for Cancer Research, Oslo University Hospital, Oslo, Norway; 2 Institute of Clinical Medicine, Faculty of Medicine, University of Oslo, Oslo, Norway; 3 Department of Pathology, Institute of Clinical Medicine, Faculty of Medicine, University of Oslo, Oslo, Norway; 4 Department of Gastrointestinal Surgery, Oslo University Hospital, Oslo, Norway

**Keywords:** Prognostic markers, Cancer microenvironment, Colorectal cancer, T cells, Immunohistochemistry

## Abstract

Regulatory T cells (Tregs) are a heterogeneous cell population that can either suppress or stimulate immune responses. Tumor-infiltrating Tregs are associated with an adverse outcome from most cancer types, but have generally been found to be associated with a good prognosis in colorectal cancer (CRC). We investigated the prognostic heterogeneity of Tregs in CRC by co-expression patterns and spatial analyses with diverse T cell markers, using multiplex fluorescence immunohistochemistry and digital image analysis in two consecutive series of primary CRCs (total *n* = 1720). Treg infiltration in tumors, scored as FOXP3^+^ or CD4^+^/CD25^+^/FOXP3^+^ (triple-positive) cells, was strongly correlated to the overall amount of CD3^+^ and CD8^+^ T cells, and consequently associated with a favorable 5-year relapse-free survival rate among patients with stage I–III CRC who underwent complete tumor resection. However, high relative expression of the activation marker CD25 in triple-positive Tregs was independently associated with an adverse outcome in a multivariable model incorporating clinicopathological and known molecular prognostic markers (hazard ratio = 1.35, *p* = 0.028). Furthermore, spatial marker analysis based on Voronoi diagrams and permutation testing of cellular neighborhoods revealed a statistically significant proximity between Tregs and CD8^+^-cells in 18% of patients, and this was independently associated with a poor survival (multivariable hazard ratio = 1.36, *p* = 0.017). These results show prognostic heterogeneity of different Treg populations in primary CRC, and highlight the importance of multi-marker and spatial analyses for accurate immunophenotyping of tumors in relation to patient outcome.

## Introduction

The immune system has been established as a central player in cancer development and progression, standing at a crossroads between pro- and anti-tumorigenic functions^1^. High densities of lymphocytes in the tumor microenvironment are associated with a good patient prognosis across several cancer types^2^. However, it has become increasingly clear that cancer cells modulate the immune microenvironment and foster anti-tumor immune evasion by a variety of mechanisms^3^. The identification of immune checkpoints and development of inhibitors against these molecules has revolutionized the treatment of certain cancers. Patients who earlier had a dismal prognosis can now experience long-term survival^4^. The microsatellite instability (MSI) phenotype predicts response to immune checkpoint inhibition, and was in 2017 the first biomarker to be approved as a treatment indication agnostic of cancer site^5^. MSI tumors generally have a dense immune infiltration^6^, associated with the high mutational burden and presentation of diverse neoantigens to the immune system.

Colorectal cancer (CRC) is responsible for a large subset of MSI tumors^7–9^. Approximately 15–20% of CRCs are of the MSI subtype^9,10^, but high levels of immune infiltration can also be found in microsatellite stable (MSS) CRCs. The Immunoscore scores pathological specimens according to the amount of infiltrating CD8^+^ and CD3^+^ lymphocytes in the invasive front and central tumor^2^, and has been extensively validated as a prognostic marker of CRC beyond the MSI phenotype^11^. However, the tumor immune contexture includes a variety of cell types beyond the CD8^+^ and CD3^+^ populations, and both the composition and organization of the total leukocyte infiltrate can have prognostic relevance^12^.

Regulatory T cells (Tregs) are a subset of T cells that play a central role in dampening the inflammatory response^13,14^. In line with this immune suppressive function, Tregs have been associated with a poor patient prognosis in several studies and across cancer types, including in a pan-cancer meta-analysis^15^. This meta-analysis also suggested that Tregs are associated with a prolonged overall survival in certain cancer types, including CRC, head and neck cancer, and esophageal cancer^15^. The choice of markers for analysis of the Treg population has been debated^16^. Traditionally, CD4^+^CD25^+^-cells have been recognized as the immune cell population with the suppressive function of Tregs^17^, but it was later demonstrated that the transcription factor FOXP3 is critical to the development of these cells^18^. FOXP3 is currently the most widely used marker to analyze Tregs by immunohistochemistry (IHC). However, expression levels of CD25 vary among FOXP3-expressing cells, and it has been shown that the subset of FOXP3^+^-cells with the highest levels of CD25 also have the phenotype with the strongest immune-suppressive activity^19^. CD25^High^-cells have been shown to suppress tumor-associated antigen responses and to be associated with recurrence also from CRC^20^. Furthermore, direct interaction with effector cells, such as cytotoxic T cells, may be needed for the Tregs to exert their immune-suppressive functions^21–23^. This implies a critical impact on the spatial context of lymphocyte populations in the tumor microenvironment, but this has not been evaluated in large series of CRCs.

We hypothesized that tumor immunophenotyping with multiple Treg markers and spatial analysis relative to markers of cytotoxic T cells would improve the prediction of outcome from CRC. Tumor immunophenotyping was performed by fluorescence-based multiplex IHC and digital image analyses of tissue microarrays (TMA) of two single-hospital series of primary CRCs (total *n* = 1720).

## Materials and methods

This study follows the Reporting recommendations for tumor marker prognostic studies (REMARK)^24^ (Supplementary Table 1).

### Patients and tumor tissue samples

Two independent, single-hospital series of primary tumor samples from patients treated by major surgical resection for stage I-IV CRC at Oslo University Hospital, Norway were analyzed on TMAs. Samples for the Norwegian series 1 were collected between 1993 and 2003 (*n* = 922), and samples for the Norwegian series 2 between 2003 and 2012 (*n* = 798). Patients were treated with adjuvant chemotherapy according to national guidelines, as previously described^25^. All tumor samples were retrospectively retrieved from the diagnostic biobank at Oslo University Hospital, and construction of TMAs from formalin-fixed paraffin-embedded (FFPE) tumor material has previously been described, with tumor tissue cores of 0.6 mm and 1.0 mm in diameter on the Norwegian Series 1 and 2 TMAs, respectively^25^. Briefly, all available tumor blocks from each patient were assessed by an expert pathologist (A. Svindland) and a representative block with tissue from the central tumor region was selected as the donor block. The recipient blocks (TMA) included a single tissue core from each patient, taken from an area of the donor block marked as representative by the pathologist. Mucinous tumors were included in the TMAs (*n* = 19 of the total 1720 samples) and in the analysis of T cells, as long as they met the technical requirements outlined in Supplementary Fig. 1. The two series have previously been analyzed for MSI status by PCR-based analysis of five microsatellite loci (BAT25, BAT26, D2S123, D5S346, and D17S250), as well as *KRAS* (exons 2 and 3) and *BRAF*^V600E^ mutation hotspots^26–28^ in DNA extracted from FFPE material and parallel fresh frozen tissue samples for series 1 and 2, respectively. MSI and *BRAF*^V600E^ mutation status based on PCR were available for 1179 and 1114 patients, respectively. IHC was performed to increase the number of samples with known MSI and *BRAF*^V600E^ mutation status. MSI status was determined by IHC in the Norwegian series 2 using antibodies targeting the four mismatch repair proteins MLH1 (clone ES05, DAKO/Agilent, Santa Clara, CA, USA), MSH2 (clone FE11, DAKO/Agilent), MSH6 (clone EP49, DAKO/Agilent), and PMS2 (clone EP51, DAKO/Agilent)^25^. BRAF^V600E^ expression was analyzed in both series using the Ventana anti-BRAF V600E (clone VE1) mouse monoclonal antibody (Roche, Basel, Switzerland). Concordance between IHC and PCR-based results for MSI status and *BRAF*^V600E^ mutation status was 97% and 96% in the 315 and 750 tumors with both data types available, respectively; the results from PCR analyses were used in cases of discrepancy.

### Multiplex immunohistochemistry

Two five-color multiplex IHC stains were performed on 4μm thick sections of the TMAs. Stain 1 (pan/cytotoxic T cell stain) was performed using antibodies against CD56 (clone MRQ-42, Cell Marque, Rocklin, CA, USA), CD8 (clone C8/144B, DAKO/Agilent), CD3 (clone F7.2.38, DAKO/Agilent), and a cocktail of antibodies targeting the epithelial cancer cells (E-cadherin clone 36 (BD-Biosciences, Franklin Lakes, NJ, USA)/cytokeratin C-11 (Abcam, Cambridge, UK)/cytokeratin Type I/II clone AE1/AE3 (Thermo Fisher Scientific, Waltham, MA, USA)). Notably, data on CD56 (neural lineage marker expressed on a subset of immune cells, including natural killer cells) was not analyzed in the present study. Stain 2 (Treg stain) was performed with antibodies against CD4 (clone EP204, Cell Marque), FOXP3 (clone D6O8R, Cell Signaling Technology, Danvers, MA, USA), CD25 (clone EP218, Cell Marque), and CD8 (clone C8/144B, DAKO/Agilent). Both stains also included incubation with DAPI for staining of cell nuclei prior to mounting. A detailed description of the antibodies and reagents used is provided in Supplementary Table 2. The stains were carried out using a four-plex kit (NEL810001KT, PerkinElmer/Akoya, Marlborough, MA, USA), together with Opal 620 reagent (FP1495001KT, PerkinElmer/Akoya) as previously described^29^. The Opal protocol (PerkinElmer/Akoya) was followed with the exception that slide deparaffinization, antigen retrieval, and antibody stripping were all performed in a PT-link module (DAKO/Agilent, Santa Clara, CA, USA) at 97 °C for 20 min. All primary antibodies were incubated for 30 min at room temperature. For stain 1, CD56 was visualized with Opal 620, CD8 with Opal 520, CD3 with Opal 570, and the cocktail targeting the epithelial cancer cells with Opal 690. For stain 2, CD4 was visualized with Opal 520, FOXP3 with Opal 690, CD25 with Opal 620, and CD8 with Opal 570. The staining process is outlined in Supplementary Table 3. Briefly, this protocol involves cycles of antibody hybridization and staining, followed by removal of the bound antibodies. The fluorescent probes bind covalently to the tissue, and several stains can be performed on the same tissue section prior to mounting and imaging. Determination of optimal concentrations of the antibodies for staining (Supplementary Table 2), and verification of complete antibody removal between staining cycles were performed in separate experiments on test-TMAs prior to staining of Norwegian series 1 and 2 (data not included).

### Digital image analysis

Stained TMAs were multispectral imaged using the Vectra 3.0 system (PerkinElmer/Akoya). A single 20× (0.5 μm/pixel) image was taken for each sample of the Norwegian Series 1 TMA (0.6 mm diameter cores). A 2 × 2 image field was captured for each sample of the Norwegian Series 2 TMA (1.0 mm diameter cores). Samples were spectrally unmixed, including removal of tissue autofluorescence, and analyzed in inForm software (PerkinElmer/Akoya). Two batch-analysis algorithms were built based on a subset of the TMA samples, one for each stain. Samples were randomly selected and used to optimize each algorithm, until the algorithms were found to be sufficiently generalizable upon addition of new images (~20 samples were sufficient for algorithm development). The algorithms were then applied to the full sample sets in batch-analysis mode. For stain 1, samples were segmented into stromal and epithelial cancer cell regions based on staining by the epithelial cocktail (empty glass was used to segment out the background). For stain 2 (no epithelial markers), the whole tissue cores were segmented from the background. For both stains, individual nuclei were segmented using signal from DAPI, while segmentation of the cell membrane and cytoplasm was aided by signals from the other markers. Following batch analysis of the two TMA-cohorts, images were manually checked and regions to be excluded from analysis (including tissue folds and necrotic regions) were marked. Marked images were submitted to re-analysis. Samples with inadequate technical quality were removed from downstream analyses (Supplementary Fig. 1).

### Marker scoring

Data tables with raw mean fluorescence intensity values per marker per cell for each TMA core and spatial coordinates per cell for each TMA core, were exported from inForm and further processed in R (v. 3.6.3). For stain 1, each individual cell in each individual TMA core was scored as positive/negative for CD3 and CD8 based on the mean signal intensity of their corresponding fluorophores. Cancer cell and area fractions were also calculated based on this stain, by dividing the number of cells in the epithelial cancer tissue region by the total number of cells, and by dividing the area occupied by the epithelial cancer tissue region by the total area, respectively. For stain 2, each individual cell was scored as positive/negative for CD4, CD25, FOXP3, and CD8. Thresholds for each marker were set within each stain and patient series individually, based on visual inspection of the images and the intensity distributions of the markers (Supplementary Fig. 2). CD8 analyzed in stain 1 showed good concordance with CD8 analyzed in stain 2 (Pearson’s *r* = 0.92 in Norwegian series 2; Supplementary Fig. 3, and 0.76 in Norwegian series 1; Supplementary Fig. 4). A lower concordance in Norwegian series 1 is expected due to the smaller size of the TMA cores. An infiltration score for each immune cell marker was calculated for each sample (tissue core) as the number of positive cells/mm^2^. For stain 1, scores were also calculated for the intraepithelial and stromal regions separately. Infiltration scores were log2 transformed (after addition of a constant of 1 for inclusion of samples with 0 positive cells) since the distributions across samples were heavily right-skewed (data not shown). Tumors were grouped by the median log2 scores of CD3 and CD8 across the sample sets, in line with previous studies^30^, while determination of thresholds delineating high/low infiltration of other cell populations is indicated where applicable. Tregs were scored both as a FOXP3^+^ cell population and as a CD4^+^/CD25^+^/FOXP3^+^ (triple-positive) cell population. The triple-positive Tregs were categorized into four groups based on high and low FOXP3 and CD25 expression, using the 75th percentile of positive cells as a threshold for both markers. Triple-positive Tregs with high levels of both markers (CD4^+^CD25^High^FOXP3^High^) represented 16% of the total triple-positive Treg population. The mean expressions of FOXP3 and CD25 across all triple-positive cells per sample were also calculated.

### Gene expression-based estimation of tumor-infiltrating immune cells

A subset of the tumors (parallel fresh frozen samples) has previously been analyzed for gene expression on the Human Exon 1.0 ST (*n* = 187) and Human Transcriptome 2.0 arrays (*n* = 167, Affymetrix Inc., Santa Clara, CA, USA)^31^. Background correction, quantile normalization, and gene-level probe summarization of the raw data CEL files were performed using the robust multi‐array average method^32^ implemented in the R package *Affy* (v 1.58.0)^33^, using custom Entrez CDF files (v22) from Brainarray^34^. Gene expression data generated on the two different platforms were merged by Entrez IDs and adjusted by batch correction using the *ComBat* function implemented in the R package *sva* (v 3.28.0)^35^. Entrez IDs were converted to HGNC gene symbols using the org.Hs.eg.db package (v 3.8.0) from Bioconductor^36^.

Gene expression-based scores for Tregs in each tumor were estimated with the algorithms from Danaher et al.^37^, CIBERSORTx^38^ and ImmuCellAI^39^, and used to determine the concordance with IHC-based estimates. Danaher et al. estimated Treg scores as the log2 expression of the single marker gene *FOXP3*. For CIBERSORTx, abundances of immune cell populations were calculated using the online platform (https://cibersortx.stanford.edu/) in high-resolution mode with 100 permutations and in absolute mode with the default LM22 mixture file. Gene expression values on log2 scale were exponentially transformed to the linear scale using the *exp* function in the base R package for input to CIBERSORTx. ImmuCellAI was run via the online platform (http://bioinfo.life.hust.edu.cn/ImmuCellAI) using the “Immune cell abundance in sample” mode.

### Spatial analysis

Spatial analysis of markers on TMAs was performed similarly to the approach described by Enfield et al.^40^. Tissue segmentation maps were exported from inForm software and used to construct tissue outlines in R software using the packages *EBImage* (v 4.28.1) and *raster* (v 3.4–5). Cell nuclei coordinates of all cells within each sample, obtained from the image analysis in inForm described above, and the tissue outlines were used to construct Voronoi diagrams with the R packages *ggvoronoi* (v 0.8.3), *sp* (v 1.4–4), *spdep* (v 1.1–5), and *rgeos* (v 0.5–5). These diagrams were then used to determine cellular neighborhoods for each individual cell. First, cells were placed into one of the following four categories based on marker expression: CD8^+^, FOXP3^+^, triple-positive Treg (CD4^+^/CD25^+^/FOXP3^+^), or other. Then, the numbers of CD8^+^-cells neighboring at least one FOXP3^+^-cell and CD8^+^-cells neighboring at least one triple-positive Treg cell were determined based on output from the *voronoi_polygon* function in the *ggvoronoi* package and the *poly2nb* function in the *spdep* package. To assess whether the neighbor frequencies of these cell populations were higher than expected by chance, permutation testing was performed by Monte Carlo sampling (*n* = 2000 permutations) within each individual tumor sample. In each permutation, cell positions and the number of cells in each of the four categories were fixed based on the observed data, while the category of each individual cell was randomized in the cell map (output from the *voronoi_polygon* function) using the function *sample* in the base R package. The numbers of CD8^+^-cells neighboring at least one FOXP3^+^-cell or triple-positive Treg were determined in each randomized cell map, and the 2000 random cell maps represented the permuted distribution of cell neighbors. The sample was classified with a significant interaction between the cell types investigated if the observed number of cell neighbors was higher than the 95th percentile of the permuted distribution (illustrated in Fig. 3C). Samples without CD8^+^-cells (*n* = 56) were not included for spatial analysis, while samples without FOXP3^+^-cells (*n* = 75) or triple-positive Tregs (*n* = 212) were classified in the non-significant interaction subgroup.

For comparison, spatial interactions were also calculated based on the fraction of CD8^+^-cells neighboring at least one FOXP3^+^-cell or triple-positive Treg per sample. However, these estimates were strongly associated with the absolute numbers of marker positive cells in each sample (Supplementary Fig. 5). The scoring of significant spatial proximities based on Monte-Carlo permutations was less influenced by the number of marker positive cells, illustrated by a more even distribution of samples with significant proximities relative to the absolute abundances of CD8^+^-cells and Tregs per sample (Fig. 3D), and was chosen for further analyses.

### Survival analyses

Survival analyses included 1316 of the totally 1720 patients, after exclusion of patients based on technical data quality (*n* = 276) and clinicopathological parameters (*n* = 128; Supplementary Fig. 1). For patients with stage I-III CRC, only those with a negative resection margin >1 mm (R0 status) were included. Patients with stage IV CRC were analyzed separately. Patients with synchronous primary CRCs at diagnosis, and those treated with pre-operative radiotherapy were excluded. Among patients with stage I–III CRC and R0 status, there were no significant differences in the clinicopathological or molecular marker distributions between included and excluded cases, with the exception of a more frequent exclusion of *BRAF* wild-type tumors (Supplementary Table 4). Five-year relapse-free survival (RFS) was evaluated as endpoint for stage I–III CRC, and calculated as time from surgery to recurrence or death from any cause, as defined by Punt et al.^41^. Overall survival was calculated as time from surgery to death from any cause and evaluated as endpoint in stage IV patients. Uni- and multivariable Cox proportional hazards models were estimated using the *coxph* function in the *survival* R package (v. 2.44.1.1), and the assumption of proportional hazards was tested using the *cox.zph* function. Kaplan-Meier plots were made using the *survminer* package (v. 0.4.6).

For the best possible comparison with the Immunoscore, the TMA cores were evaluated for combinations of CD3 and CD8 scores in the intraepithelial and stromal tissue compartments. The optimal marker combination for prediction of 5-year RFS was evaluated by the Akaike Information Criterion. Potentially confounding variables included in multivariable analysis of CD3 and CD8 (Table 2) were the same as used in the international validation of the Immunoscore^11^; pT and pN stage, MSI status, patient age and sex. Notably, this study included both colon and rectal cancers, and tumor location was therefore added as a variable. Multivariable survival analyses were stratified by cohort (Norwegian Series 1 and 2).

For exploratory analyses of Tregs, Norwegian Series 2 served as a discovery series due to its larger TMA-cores, providing more robust estimation of tumor-infiltrating immune cells per sample. Norwegian Series 1 served as a historical validation series. For the final multivariable survival model (Table 3), three variables involving Tregs (CD25-expression in triple-positive Tregs, spatial associations between CD8^+^-cells and FOXP3^+^-cells and spatial associations between CD8^+^-cells and triple-positive Tregs) and *BRAF*^V600E^ status were considered together with the variables included for evaluation of CD3 and CD8 scores, described above. Final selection of variables was performed by bootstrap sampling (*n* = 1000) combined with a backward selection procedure implemented in the *mfp* package (v. 1.5.2), in line with the methodology described in Sauerbrei and Schumacher^42^. This procedure indicated a nonsignificant prognostic distinction between pT stage 1 and 2, and the two subcategories were therefore combined in the final model. Variables that were retained in less than 30% of the runs were excluded from the final model. Patients were excluded from multivariable analysis if data was missing for any of the variables included. *KRAS* mutation status was missing in 41% of cases and was only used to test for associations with the immune cell scores, and not included in multivariable survival analyses.

### Statistics

All statistical analyses were performed in RStudio v. 1.1.383 with R v. 3.6.3. All statistical tests were two-sided (except for the determination of spatial proximity, which was one-sided) and *p* values less than 0.05 were considered significant. Infiltration scores of different immune cell populations were evaluated both as continuous variables and after dichotomization into high/low categories. The correlation-matrices for immune cell populations were based on Pearson’s correlations and produced using the *corrplot* package (v. 0.84).

## Results

### Multiplexed analysis of CD3 and CD8 on tissue microarrays recapitulates the prognostic value of T cells

**Fig. 1:**
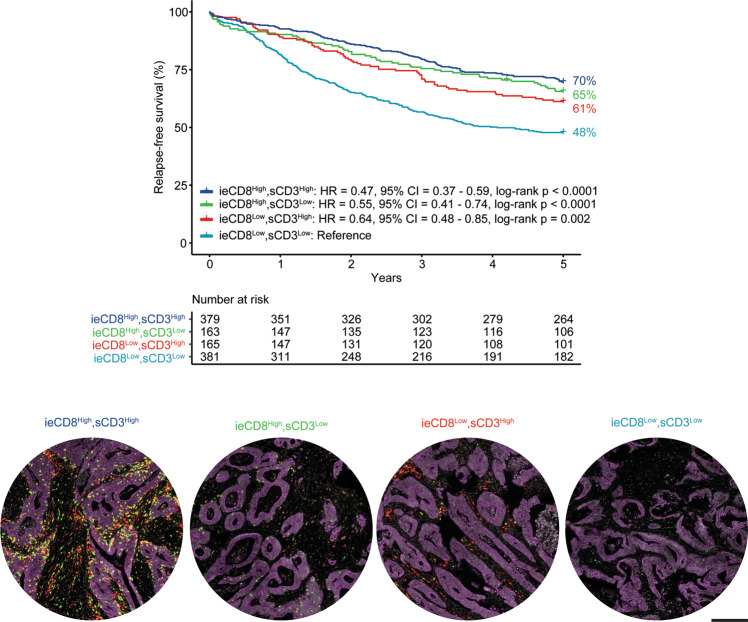
Infiltrating lymphocytes, scored by tissue-microarray analysis, are strongly associated with CRC patient prognosis. Intraepithelial (ie) CD8- and stromal (s) CD3-scores were dichotomized at the median within the pooled cohorts, resulting in four groups; ieCD8^High^sCD3^High^, ieCD8^High^sCD3^Low^, ieCD8^Low^sCD3^High^ and ieCD8^Low^sCD3^Low^. Representative images of tissue-cores for each of these categories are displayed (red; CD3, green; CD8, purple; epithelial (tumor) markers, white; DAPI). Scale-bar equals 200μm.

Immune marker infiltration scores per mm^2^ tumor tissue in the two CRC series (*n* = 1720) are presented in Table 1, together with clinicopathological and molecular characteristics. Stromal and intraepithelial scores of CD3 and CD8 were correlated (Pearson’s correlation 0.52–0.77; Supplementary Figs. 3 and 4) and univariable survival analyses demonstrated that both markers in both tissue compartments were strongly associated with 5-year RFS among patients treated by complete resection for stage I–III CRC (*n* = 1088; Supplementary Tables 5 and 6). Akaike’s information criterion indicated that the optimal prognostic combination of markers was intraepithelial CD8 and stromal CD3 (Supplementary Tables 5 and 6), and patients with a low expression of both had the worst survival (Fig. 1). Both intraepithelial CD8 and stromal CD3 infiltration were inversely associated with depth of tumor infiltration (pT) and nodal involvement (pN), specifically in MSS cancers (Supplementary Table 7). Both markers retained prognostic value in multivariable survival models when analyzed as categorical (Table 2) and/or continuous variables (Supplementary Table 8), but with strongest statistical significance for intraepithelial CD8. A stratified analysis indicated that MSI status was associated with a favorable patient survival only in the subgroup with low immune cell scores (low intraepithelial CD8 and stromal CD3), and this was not attributed to differences in the immune cell densities between the MSI and MSS tumors (Supplementary Fig. 6).

**Table 1:** Clinicopathological and molecular characteristics of the two series.

		Norwegian series 1 (1993–2003)	Norwegian series 2 (2003–2012)	*p* value
Total patients, *n*		922	798	
Age	Median (range)	73 (29–94)	72 (27–97)	0.60
Sex	Female	485 (53%)	407 (51%)	0.53
	Male	437 (47%)	391 (49%)	
TNM stage	I	137 (15%)	167 (21%)	0.007
	II	381 (41%)	288 (36%)	
	III	242 (26%)	214 (27%)	
	IV	159 (17%)	129 (16%)	
	NA	3	–	
pT	1	37 (4%)	40 (5%)	0.0007
	2	127 (14%)	165 (21%)	
	3	662 (72%)	515 (65%)	
	4	96 (10%)	72 (9%)	
	NA	0	6	
pN	0	563 (62%)	491 (62%)	0.0008
	1	250 (27%)	175 (22%)	
	2	99 (11%)	129 (16%)	
	NA	10	3	
Residual tumor (R) status	R0	719 (78%)	651 (82%)	0.09
	R1	36 (4%)	19 (2%)	
	R2	167 (18%)	128 (16%)	
Tumor location	Right colon	365 (40%)	327 (41%)	0.29
	Left colon	301 (33%)	239 (30%)	
	Rectum	231 (25%)	218 (27%)	
	Synchronous	25 (3%)	14 (2%)	
MSI status	MSI	128 (15%)	120 (16%)	0.78
	MSS	712 (85%)	638 (84%)	
	NA	82	40	
*BRAF*^V6ooE^ mutational status	Wild-type	714 (85%)	637 (84%)	0.63
	Mutated	127 (15%)	122 (16%)	
	NA	81	39	
*KRAS* mutational status	Wild-type	463 (69%)	238 (69%)	0.94
	Mutated	204 (31%)	106 (31%)	
	NA	255	454	
Adjuvant chemotherapy	No	806 (87%)	609 (79%)	<0.0001^b^
	Yes	116 (13%)	162 (21%)	
	NA	0	27	
Total CD3^+^-cells per mm^2^ (log2)^a^	Median (Q1–Q3)	8.8 (7.3–10.0)	8.8 (7.3–9.9)	0.46
Total CD8^+^-cells per mm^2^ (log2)^a^	Median (Q1–Q3)	6.1 (4.2–8.0)	6.0 (4.3–7.5)	0.41
Total FOXP3^+^-cells per mm^2^ (log2)^a^	Median (Q1–Q3)	5.9 (3.9–7.4)	5.8 (4.4–7.1)	0.76
Total tp-Treg-cells per mm^2^ (log2)^a^	Median (Q1–Q3)	3.8 (0–5.4)	3.6 (1.9–5.0)	0.82

*p* values were calculated to determine if there were any statistical differences between the two consecutive Norwegian series; Wilcoxon rank-sum test was used for age and the immune-cell scores, Fisher Exact test for sex, adjuvant chemotherapy, MSI-, *BRAF-* and *KRAS-*status and chi-squared test for TNM stage, pT, pN, residual tumor status and tumor location.

*MSI* microsatellite instable, *MSS* microsatellite stable, *pN* regional lymph node classification, *pT* primary tumor classification, *TNM* Tumor-node-metastasis, *tp-Treg* triple-positive Treg.

^a^*n*(Norwegian series 1) = 757 and *n*(Norwegian series 2) = 687; including all samples that were not excluded due to technical reasons (Supplementary Fig. 1).

^b^National guidelines for adjuvant treatment have changed over time, reflecting the increased proportion receiving such therapy in the Norwegian series 2. Adjuvant chemotherapy for stage III CRC was introduced in national guidelines in 1997.

**Table 2:** Multivariable survival analysis of intraepithelial CD8- and stromal CD3-positive T-cell scores.

	Univariable analysis			Multivariable analysis c-index (concordance): 0.693 (se = 0.013)		
Statistic	HR	95% CI	*p* value	HR	95% CI	*p* value
ieCD8						
High vs low^a^	0.55	0.45–0.67	<0.0001	0.65	0.52–0.81	0.0001
sCD3						
High vs low^a^	0.60	0.49–0.73	<0.0001	0.81	0.65–1.01	0.066
Sex						
Women vs men	0.99	0.81–1.20	0.89	0.85	0.69–1.04	0.11
Tumor location						
Left vs right	1.19	0.95–1.48	0.13	1.16	0.92–1.47	0.21
Rectum *vs* right	0.89	0.69–1.16	0.40	1.14	0.86–1.51	0.38
pT						
T2 vs T1	0.99	0.56–1.76	0.98	0.89	0.50–1.58	0.69
T3 vs T1	1.91	1.14–3.21	0.015	1.37	0.80–2.33	0.25
T4 vs T1	3.30	1.78–6.11	0.00015	2.69	1.42–5.09	0.0024
pN						
N1 vs N0	1.67	1.33–2.09	<0.0001	1.52	1.20–1.92	0.00047
N2 vs N0	2.66	1.99–3.56	<0.0001	2.44	1.81–3.29	<0.0001
MSI status						
MSI vs MSS	0.71	0.54–0.93	0.013	0.78	0.58–1.07	0.12
Age^a^	1.04	1.03–1.05	<0.0001	1.04	1.03–1.05	<0.0001

The analysis was stratified by cohort and performed within stage I–III, R0 CRC patients. Endpoint evaluated was 5-year relapse-free survival. Only patients with complete data for all variables were included in the analysis. Dichotomized CD8 and CD3 scores were used; analysis of the continuous variables is presented in Supplementary Table 8.

*n* = 1028, events = 406.

*ieCD8* intraepithelial CD8, *MSI* microsatellite instable, *MSS* microsatellite stable, *sCD3* stromal CD3.

^a^Violates proportional hazards assumption in univariable analysis.

Primary tumors from stage IV CRCs (*n* = 228) had significantly lower amounts of infiltrating T cells (Supplementary Table 9). This could not solely be attributed to the different distributions of certain clinicopathological characteristics (patient age and tumor location) or MSI status between stage IV and stage I–III cancers, and stage IV cancer remained a significant predictor of a low infiltration of the various T cell populations in linear models incorporating these characteristics (Supplementary Table 10). Interestingly, the largest difference in T cell abundances between stages I–III and IV was found within the intraepithelial compartment (Supplementary Table 10). Neither intraepithelial CD8 infiltration nor stromal CD3 infiltration were associated with 5-year overall survival in stage IV patients (Supplementary Fig. 7) and these patients were not included in further analyses.

### CD25 expression is a discriminatory feature for the prognostic value of Tregs

**Fig. 2:**
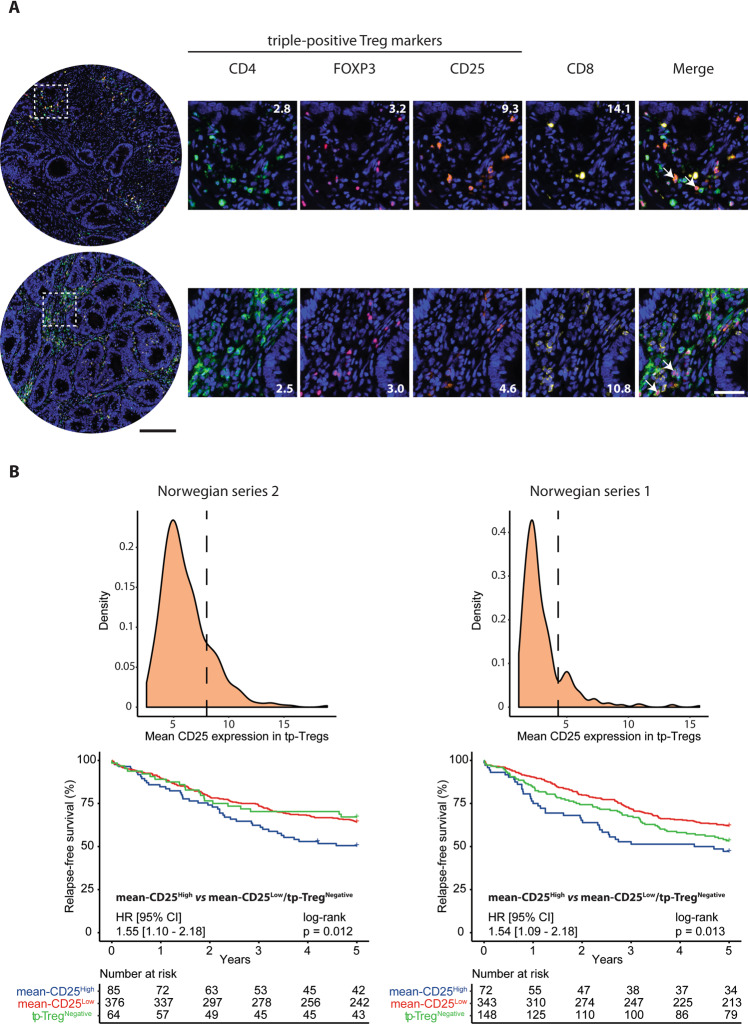
High mean expression of CD25 in triple-positive Tregs is associated with adverse prognosis in CRC. **A** Representative images of samples containing high (top) and low (bottom) levels of CD25 in triple-positive Tregs. Numbers on images represent the mean value of the marker in positive cells. Arrows in the merged images indicate examples of triple-positive Tregs. Black scale-bar equals 200μm (in the core-images) and white scale-bar equals 50 μm (in the cropped images). **B** Density plots with the cutoffs for separating samples with low- and high mean expression of CD25 in triple-positive Tregs (tp-Tregs). The cutoffs were set individually for each series, at the point that appeared as a shoulder for Norwegian series 2 and at the point between the two maxima for Norwegian series 1. Two samples with mean expression >20 were excluded from the density plot for Norwegian series 2, and one from the plot for the Norwegian series 1, for visualization purposes. Kaplan-Meier survival analysis was performed in the Norwegian series 2 (left) and Norwegian series 1 (right) individually; patients with mean-CD25^High^ samples were compared to those with mean-CD25^Low^ and those with no triple-positive Tregs (tp-Treg^Negative^) combined.

For analysis of the Treg population, we initially compared different proposed markers. There was a strong correlation among tumors in the Norwegian series 2 for IHC-scores of FOXP3 alone and the cell population identified as triple-positive for CD4/CD25/FOXP3 (Pearson’s correlation 0.85, *p* < 0.0001). Both Treg-scores were moderately correlated to the other T-cell markers (CD4, CD3, CD8; Pearson’s correlation 0.3–0.67; Supplementary Fig. 3). However, there was a large range in the mean fluorescence intensity of CD25 (10th–90th percentile = 3.9–9.1) and FOXP3 (10th–90th percentile = 1.3–4.8) within the triple-positive Treg population across samples (Fig. 2A, B, Supplementary Fig. 8A), and the two scores were not correlated (Pearson’s correlation −0.04; Supplementary Fig. 9). Mean expression of CD25 (and not FOXP3) in triple-positive Tregs was weakly negatively correlated to CD8 infiltration (*R* = −0.20, *p* < 0.0001, Supplementary Figs. 8B and 10A), while samples with no triple-positive Tregs had the lowest CD8 infiltration (median CD8^+^-cells per mm^2^ (log2) = 4.2, Supplementary Figs. 8C and 10B).

Tregs were also analyzed on the transcriptomic level in parallel fresh frozen samples from a subset of the tumors (*n* = 270), using three common algorithms for estimation of immune cell populations. There was no correlation between IHC-based FOXP3 infiltration and level of Treg infiltration estimated by the transcriptomic signatures from Danaher et al. (*FOXP3* only) or CIBERSORTx (Supplementary Fig. 11A, B, respectively), but a weak correlation with the ImmuCellAI Treg signature (Supplementary Fig. 11C). However, the ImmuCellAI signature was also correlated with IHC-based CD8 infiltration, indicating estimation of a broader immune cell population (Supplementary Fig. 12). Notably, the gene expression-based Treg signatures correlated weakly with each other (Supplementary Fig. 11D–F), while correlations for signatures of CD8^+^-cells were stronger (Supplementary Fig. 13), supporting that Tregs are a particularly difficult cell population to accurately score across methods.

High scores for the strongly correlated Treg populations estimated by IHC of FOXP3 and CD4/CD25/FOXP3 were both associated with a higher 5-year RFS rate among patients with stage I-III CRC in Norwegian Series 2 (Supplementary Fig. 14A). Neither of the Treg populations had a prognostic impact within the subgroup of patients with high intraepithelial CD8 and stromal CD3 scores, inconsistent with the hypothesis that Tregs have a negative influence on the activity of other T cell populations (Supplementary Fig. 14B). Considering the large variation in CD25 and FOXP3 expression among the triple-positive Tregs, these cells were further divided into four groups according to the expression levels of FOXP3 and CD25 (described in methods). High infiltration of CD4^+^CD25^High^FOXP3^High^-cells was also associated with a better 5-year RFS (Supplementary Fig. 15). However, analysis of CD25 by its mean intensity value among triple-positive Tregs showed that patients with a high mean CD25 expression (mean-CD25^High^) had a significantly worse prognosis than patients with a low mean CD25 expression (mean-CD25^Low^) or no triple-positive Tregs (HR = 1.55, CI = 1.10–2.18, *p* = 0.012, Fig. 2B, left). Corresponding analysis of FOXP3 expression did not reveal any prognostic associations (Supplementary Fig. 8D). The negative correlation between mean CD25 expression in triple-positive Tregs and CD8^+^-cell infiltration identified in the Norwegian series 2 was validated in the Norwegian series 1 (Supplementary Fig. 16), and patients in the Norwegian series 1 with mean-CD25^High^ samples also had inferior 5-year RFS compared to those with mean-CD25^Low^ or no triple-positive Treg samples (HR = 1.54, CI = 1.09–2.18, *p* = 0.013, Fig. 2B, right).

### Spatial proximity between Tregs and cytotoxic T cells in a subset of CRCs

**Fig. 3:**
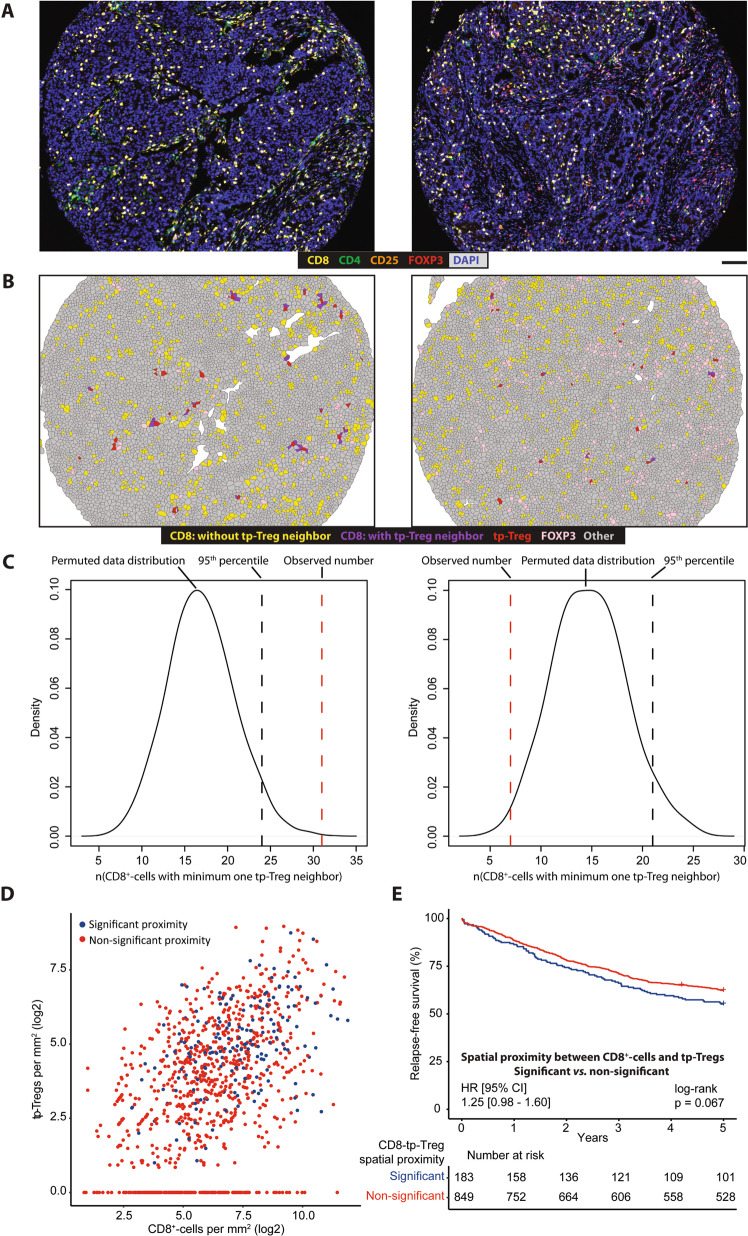
Spatial association analysis of CD8^+^-cells and triple-positive Tregs. One example of a sample displaying significant spatial proximity between CD8^+^-cells and triple-positive Tregs is shown on the left, and one displaying non-significant spatial proximity is shown on the right (**A**–**C**). **A** Spectrally unmixed composite images of the samples exported from inForm software. Scale-bar equals 100 μm in **A**, **B**. **B** Voronoi diagrams constructed in R. **C** Density plots of the permuted data distribution were used to determine whether the observed number of neighbors between cell types was higher than could be expected by chance. The sample on the left had higher amounts of CD8^+^-cells neighboring triple-positive Tregs than the 95th percentile of the permuted distribution and is therefore classified as having a significant association between the cell types. Conversely, the sample on the right had lower amounts of CD8^+^-cells neighboring triple-positive Tregs than the 95th percentile of the permuted distribution and is therefore classified as having a non-significant association. **D** Plot of CD8^+^-cell infiltration versus triple-positive Treg infiltration, colored according to whether a sample was classified as having significant spatial proximity between CD8^+^-cells and triple-positive Tregs, or not. **E** Kaplan-Meier survival analysis according to the groups defined by spatial proximity between CD8^+^-cells and triple-positive Tregs. Analysis performed within the pooled Norwegian series 1 and 2. Abbreviation: tp-Tregs; triple-positive Tregs.

Spatial proximity to CD8^+^-cells is essential for some of the immune suppressive functions of Tregs, and was initially analyzed on the TMA with largest tumor cores (Norwegian series 2; Methods; Fig. 3A–C). A statistically significant proximity between CD8^+^-cells and FOXP3^+^-cells or triple-positive Tregs was found in 47% and 30% of intraepithelial-CD8^High^/stromal-CD3^High^ tumors, respectively (Supplementary Table 11). This corresponded to 40 and 22% of the total series of stage I–III tumors with R0-status (Supplementary Table 11). Spatial proximity was more frequent in samples with high densities of CD8^+^- and FOXP3^+^-cells or triple-positive Tregs (Fig. 3D and Supplementary Fig. 17), but was not associated with MSI status (Supplementary Table 11; *p* = 0.91 and 0.50, respectively). Notably, there was no significant difference in the cancer cell fraction or cancer area fraction of tumors according to the spatial proximity groups (Supplementary Fig. 17).

Patients whose tumors had a significant proximity between CD8^+^- and FOXP3^+^-cells or between CD8^+^-cells and triple-positive Tregs had lower 5-year RFS compared to patients with CD8^+^ tumors and non-significant proximity to Tregs, although not statistically significant (Supplementary Fig. 18, top). In the Norwegian series 1, a similar prognostic association for spatial proximity was found only with the triple-positive Treg population (Supplementary Fig. 18, bottom). Data for the two series were pooled (Fig. 3E), and subgroup analyses according to T cell abundances supported the indications of a worse survival for patients with significant proximity between CD8^+^-cells and triple-positive Tregs irrespective of the level of intraepithelial CD8 and stromal CD3 infiltration (Supplementary Fig. 19).

### Tregs and spatial proximity to cytotoxic T cells are associated with poor survival in multivariable analysis

A multivariable model for prediction of 5-year RFS in the pooled series of stage I-III CRCs (*n* = 1088) was built based on the various prognostic immune cell estimates and clinicopathological factors. A high mean CD25 expression in triple-positive Tregs was found in 14% (*n* = 157) of the total tumor series (Supplementary Table 12). Mean-CD25^High^ samples were found more frequently in patients with higher pN stage (*p* = 0.005, Supplementary Table 12), and were less infiltrated by intraepithelial CD8- and stromal CD3-cells (*p* < 0.0001 for both populations, Supplementary Table 12). Significant proximity between CD8^+^-cells and triple-positive Tregs was found in 18% of tumors (*n* = 183 of the 1032 evaluated samples; *n* = 56 samples with no detected CD8^+^-cells were excluded from this analysis) (Supplementary Table 13). This measure was associated with intraepithelial CD8 (*p* = 0.0004) and stromal CD3 (*p* < 0.0001) also in the pooled patient series, but not with MSI status (*p* = 0.92) or any of the other clinicopathological features evaluated (Supplementary Table 13). Bootstrapped sampling and a backward selection procedure of the full model was implemented to determine the optimal combination of predictors (described in methods, results presented in Supplementary Table 14). Intraepithelial CD8^+^-cell infiltration, mean CD25-expression level in triple-positive Tregs and spatial proximity between CD8^+^-cells and triple-positive Tregs were all included in the final multivariable model, together with MSI status, pT- and pN-status, and patient age (Table 3 and Supplementary Tables 14 and 15). Stromal CD3^+^-cell infiltration was included in 42% of the models during bootstrapping and backward selection, but did not add independent value to the final model (Supplementary Tables 14 and 16). The model was also evaluated upon inclusion of adjuvant chemotherapy as a possible confounder, but this variable did not have a significant impact (Supplementary Table 17). All three immune cell estimates had independent prognostic value in the final model, and CD8^+^-cells were strongly associated with prolonged 5-year RFS, while each of the triple-positive Treg measures were independently associated with shorter RFS (Table 3). Notably, multivariable subgroup analyses according to MSI status indicated that proximity between CD8^+^-cells and triple-positive Tregs was prognostic only in MSI (Supplementary Table 18), while high mean expression of CD25 in triple-positive Tregs was prognostic only in MSS (Supplementary Table 19).

**Table 3:** Multivariable survival analysis of intraepithelial CD8 and triple-positive Treg scores.

	Multivariable analysis c-index (concordance): 0.695 (se = 0.013)		
Statistic	HR	95% CI	*p* value
ieCD8			
High vs low	0.62	0.50–0.76	<0.0001
Mean CD25 expression in tp-Tregs			
High *vs* Low/tp-Treg^Negative^	1.35	1.03–1.75	0.028
CD8-tp-Treg spatial proximity			
Significant *vs* non-significant	1.36	1.06–1.75	0.017
pT			
T3 vs T1/2	1.50	1.14–1.98	0.0038
T4 vs T1/2	3.00	1.93–4.64	<0.0001
pN			
N1 vs N0	1.54	1.21–1.97	0.00050
N2 vs N0	2.34	1.72–3.18	<0.0001
MSI status			
MSI vs MSS	0.72	0.54–0.96	0.027
Age	1.04	1.03–1.05	<0.0001

The variables selected by bootstrap sampling and backwards selection were included in multivariable survival analysis of the combined Norwegian series. Analysis using the continuous score for intraepithelial CD8 is presented in Supplementary Table 15 and analysis including the stromal CD3 score is presented in Supplementary Table 16. The analysis was stratified by cohort and performed within stage I–III, R0 CRC patients. Endpoint evaluated was 5-year relapse-free survival. Only patients with complete data for all variables were included in the analysis.

*n* = 977, events = 376.

*ieCD8* intraepithelial CD8, *MSI* microsatellite instable, *MSS* microsatellite stable, *tp-Tregs* triple-positive Tregs.

## Discussion

This study presents a detailed multi-marker analysis of Tregs in primary CRC, and shows that both the expression level of CD25 in CD4/CD25/FOXP3 triple-positive Tregs and spatial proximity to cytotoxic T cells are key factors to understand the prognostic impact of these immune cells. Tregs are a heterogeneous cell population that can both stimulate and suppress immune responses depending on their phenotype and marker expressions^16^. It has been indicated that Tregs have T-cell-suppressive functions in CRC^20,43^, but prognostic studies have shown that high amounts of tumor-infiltrating Tregs are associated with an improved patient outcome^15,44,45^. This is apparently contradictory to the thoroughly validated favorable prognosis associated with infiltrating CD3^+^ and CD8^+^ T cells in CRC^11^. This study supported the favorable prognosis associated with both the cytotoxic and regulatory T cell populations, possibly attributed to their strong correlation in infiltration densities among tumors. Furthermore, Tregs identified both by FOXP3 expression alone and in combination with CD4 and CD25 (triple-positive) showed similar results, again associated with a strong correlation in densities of the two immune populations among tumors, and indicating that the choice of markers does not confound the analysis.

However, subsets of FOXP3^+^- and/or CD25^+^-cells can have very different functions depending on their expression levels and the specific set of co-expressed molecules^16^. Our analyses showed that the large variation in CD25-expression in triple-positive Tregs was both inversely correlated to CD8^+^-T cell infiltration and strongly prognostic, with a high CD25 expression level apparently conferring a negative impact of Tregs on patient survival. Expression of CD25 on Tregs can deprive other T cells of IL-2, a cytokine with anti-apoptotic effects^46^. Furthermore, suppressive Tregs can also promote the destruction of T cells and exert other negative influences on surrounding immune cells^21^. The subpopulation of immune suppressive Tregs is therefore a potential drug target, and anti-CD25 antibodies have been shown to synergize with anti-PD1 treatment in mouse models transplanted with CRC cell lines^47^. Previous versions of anti-CD25 antibodies have failed to provide clinical responses against solid cancers, which can be explained by a negative bystander effect on IL-2 receptor signaling in effector T cells^48^. New anti-CD25 antibodies have been optimized to selectively deplete Tregs, while preserving IL-2 signaling in other effector T cells, and have shown exciting results in pre-clinical models^48^. Another potential strategy to inhibit the function of Tregs in the tumor microenvironment is through treatment with COX inhibitors^43^.

The spatial relationship with CD8^+^-cells was found to be another discriminatory factor in the prognostic evaluation of Tregs, consistent with exertion of immune suppressive functions via direct cell-cell interactions^21^. Due to the exploratory nature of these analyses, our study could not disentangle a separate impact of CD25 expression and spatial proximity with cytotoxic T cells on the prognostic value of Tregs. However, both Treg estimates showed independent prognostic value in multivariable models. Subgroup analyses also indicated a dependence on MSI status. A prognostic impact of spatial proximity primarily in MSI tumors may reflect the higher overall level of immune infiltrates in this subtype, while an impact of CD25 expression levels primarily in MSS may reflect a more subtle functional effect associated with this marker. Independent studies are needed to validate these associations. This study is the first to analyze the in situ proximity of cytotoxic and regulatory T cells in relation to patient outcome in a large series of CRCs. We adapted a previously published approach^40^ to determine the statistical rigor of marker positive cell neighbors by Monte-Carlo simulations. Tumor epithelial markers were not included in the stains used to analyze Tregs in association with CD8^+^-cells. Accordingly, we did not take into account a potential difference in the spatial distribution of the immune cells between tumor epithelial and stromal regions. However, using data obtained from the neighboring tissue section (i.e., stain 1 described in Supplementary Table 3), no significant difference in cancer cell fractions were found in the samples determined to have significant vs nonsignificant neighbor associations between CD8^+^-cells and triple-positive Tregs.

Gene expression profiles of neighboring tumor tissue samples supported the notion that Tregs are particularly difficult to score. Correlations of Treg scores from three different algorithms for estimation of immune cell abundances were relatively poor, and weaker than for scoring of cytotoxic lymphocytes. Correspondence with IHC-based Treg estimates were also poor, including for FOXP3 expression on the gene and protein levels, although this could be attributed to intra-tumor heterogeneity resulting from analyses of parallel tumor samples.

A limitation of this study is the analysis of tissue cores from the central tumor region rather than whole-tissue sections. A formal comparison with the Immunoscore could therefore not be made, although we noted that the intraepithelial CD8 and stromal CD3 scores showed a similar prognostic power in multivariable models compared to separate reports on the Immunoscore in stage I–III colon cancer^11^. Use of TMAs facilitated a higher throughput for multi-marker analyses, and illustrated the potential for improved prognostic stratification of CRCs beyond the subgroups defined by CD8^+^ and CD3^+^ T cells. Analysis of Tregs according to CD25 expression and spatial associations with cytotoxic T cells refined the prognostic value of immune cell scoring in colorectal cancer, indicating the need for a more detailed tumor immunophenotyping in relation to patient outcome.

In conclusion, we have demonstrated heterogeneity in the prognostic associations of different Treg populations in primary CRC by multiplex immunohistochemistry and spatial marker analysis. A high total abundance of Tregs is associated with a favorable patient outcome, but triple-positive Tregs with a high mean expression of CD25 or spatial proximity to CD8^+^-cells are independent markers of a poor prognosis. If validated in independent studies, these results could serve as grounds to explore the use of anti-CD25 antibodies to improve anti-tumor immunity in a subset of CRC patients.

## Supplementary information

Supplementary material

## Data Availability

The data in this study are available from the corresponding author upon reasonable request. Only limited clinical data for *individual* patients can be shared due to national legislation regarding privacy protection.

## References

[1] Hanahan D, Weinberg RA. Hallmarks of cancer: the next generation. *Cell* **144**, 646–74 (2011). 10.1016/j.cell.2011.02.013 21376230

[2] Bruni D, Angell HK, Galon J. The immune contexture and Immunoscore in cancer prognosis and therapeutic efficacy. *Nat Rev Cancer* **20**, 662–80 (2020). 10.1038/s41568-020-0285-7 32753728

[3] Vinay DS, Ryan EP, Pawelec G, Talib WH, Stagg J, Elkord E, et al. Immune evasion in cancer: Mechanistic basis and therapeutic strategies. *Semin Cancer Biol* **35** Suppl, S185–S98 (2015). 10.1016/j.semcancer.2015.03.004 25818339

[4] Ribas A, Wolchok JD. Cancer immunotherapy using checkpoint blockade. *Science* **359**, 1350–5 (2018). 10.1126/science.aar4060 PMC7391259 29567705

[5] Lemery S, Keegan P, Pazdur R. First FDA Approval Agnostic of Cancer Site — When a Biomarker Defines the Indication. *N Engl J Med* **377**, 1409–12 (2017). 10.1056/NEJMp1709968 29020592

[6] Llosa NJ, Cruise M, Tam A, Wicks EC, Hechenbleikner EM, Taube JM, et al. The vigorous immune microenvironment of microsatellite instable colon cancer is balanced by multiple counter-inhibitory checkpoints. *Cancer Discov* **5**, 43–51 (2015). 10.1158/2159-8290.CD-14-0863 PMC4293246 25358689

[7] Lothe RA, Peltomaki P, Meling GI, Aaltonen LA, Nystrom-Lahti M, Pylkkanen L, et al. Genomic instability in colorectal cancer: relationship to clinicopathological variables and family history. *Cancer Res* **53**, 5849–52 (1993). 8261392

[8] Peltomaki P, Lothe RA, Aaltonen LA, Pylkkanen L, Nystrom-Lahti M, Seruca R, et al. Microsatellite instability is associated with tumors that characterize the hereditary non-polyposis colorectal carcinoma syndrome. *Cancer Res* **53**, 5853–5 (1993). 8261393

[9] Bonneville R, Krook MA, Kautto EA, Miya J, Wing MR, Chen HZ, et al. Landscape of microsatellite instability across 39 cancer types. *JCO Precis Oncol* **2017**, 1–15 (2017). 10.1200/PO.17.00073 PMC5972025 29850653

[10] Grady WM, Carethers JM. Genomic and epigenetic instability in colorectal cancer pathogenesis. *Gastroenterology* **135**, 1079–99 (2008). 10.1053/j.gastro.2008.07.076 PMC2866182 18773902

[11] Pages F, Mlecnik B, Marliot F, Bindea G, Ou FS, Bifulco C, et al. International validation of the consensus Immunoscore for the classification of colon cancer: a prognostic and accuracy study. *Lancet* **391**, 2128–39 (2018). 10.1016/S0140-6736(18)30789-X 29754777

[12] Fridman WH, Zitvogel L, Sautes-Fridman C, Kroemer G. The immune contexture in cancer prognosis and treatment. *Nat Rev Clin Oncol* **14**, 717–34 (2017). 10.1038/nrclinonc.2017.101 28741618

[13] Sakaguchi S. Naturally arising CD4+ regulatory t cells for immunologic self-tolerance and negative control of immune responses. *Annu Rev Immunol* **22**, 531–62 (2004). 10.1146/annurev.immunol.21.120601.141122 15032588

[14] Vignali DA, Collison LW, Workman CJ. How regulatory T cells work. *Nat Rev Immunol* **8**, 523–32 (2008). 10.1038/nri2343 PMC2665249 18566595

[15] Shang B, Liu Y, Jiang SJ, Liu Y. Prognostic value of tumor-infiltrating FoxP3+ regulatory T cells in cancers: a systematic review and meta-analysis. *Sci Rep* **5**, 15179 (2015). 10.1038/srep15179 PMC4604472 26462617

[16] Togashi Y, Shitara K, Nishikawa H. Regulatory T cells in cancer immunosuppression—implications for anticancer therapy. *Nat Rev Clin Oncol* **16**, 356–71 (2019). 10.1038/s41571-019-0175-7 30705439

[17] Sakaguchi S, Sakaguchi N, Asano M, Itoh M, Toda M. Immunologic self-tolerance maintained by activated T cells expressing IL-2 receptor alpha-chains (CD25). Breakdown of a single mechanism of self-tolerance causes various autoimmune diseases. *J Immunol* **155**, 1151–64 (1995). 7636184

[18] Fontenot JD, Gavin MA, Rudensky AY. Foxp3 programs the development and function of CD4+CD25+ regulatory T cells. *Nat Immunol* **4**, 330–6 (2003). 10.1038/ni904 12612578

[19] Miyara M, Yoshioka Y, Kitoh A, Shima T, Wing K, Niwa A, et al. Functional delineation and differentiation dynamics of human CD4+ T cells expressing the FoxP3 transcription factor. *Immunity* **30**, 899–911 (2009). 10.1016/j.immuni.2009.03.019 19464196

[20] Betts G, Jones E, Junaid S, El-Shanawany T, Scurr M, Mizen P, et al. Suppression of tumour-specific CD4+ T cells by regulatory T cells is associated with progression of human colorectal cancer. *Gut* **61**, 1163–71 (2012). 10.1136/gutjnl-2011-300970 PMC3388728 22207629

[21] Sakaguchi S, Yamaguchi T, Nomura T, Ono M. Regulatory T cells and immune tolerance. *Cell* **133**, 775–87 (2008). 10.1016/j.cell.2008.05.009 18510923

[22] Feichtenbeiner A, Haas M, Buttner M, Grabenbauer GG, Fietkau R, Distel LV. Critical role of spatial interaction between CD8(+) and Foxp3(+) cells in human gastric cancer: the distance matters. *Cancer Immunol Immunother* **63**, 111–9 (2014). 10.1007/s00262-013-1491-x PMC11029441 24170095

[23] Posselt R, Erlenbach-Wunsch K, Haas M, Jessberger J, Buttner-Herold M, Haderlein M, et al. Spatial distribution of FoxP3+ and CD8+ tumour infiltrating T cells reflects their functional activity. *Oncotarget* **7**, 60383–94 (2016). 10.18632/oncotarget.11039 PMC5312390 27494875

[24] McShane LM, Altman DG, Sauerbrei W, Taube SE, Gion M, Clark GM, et al. Reporting recommendations for tumor marker prognostic studies (REMARK). *J Natl Cancer Inst* **97**, 1180–4 (2005). 10.1093/jnci/dji237 16106022

[25] Bergsland CH, Bruun J, Guren MG, Svindland A, Bjornslett M, Smeby J, et al. Prediction of relapse-free survival according to adjuvant chemotherapy and regulator of chromosome condensation 2 (RCC2) expression in colorectal cancer. *ESMO Open* **5**, e001040 (2020). 10.1136/esmoopen-2020-001040 PMC7682466 33219056

[26] Merok MA, Ahlquist T, Royrvik EC, Tufteland KF, Hektoen M, Sjo OH, et al. Microsatellite instability has a positive prognostic impact on stage II colorectal cancer after complete resection: results from a large, consecutive Norwegian series. *Ann Oncol* **24**, 1274–82 (2013). 10.1093/annonc/mds614 PMC3629894 23235802

[27] Vedeld HM, Merok M, Jeanmougin M, Danielsen SA, Honne H, Presthus GK, et al. CpG island methylator phenotype identifies high risk patients among microsatellite stable BRAF mutated colorectal cancers. *Int J Cancer* **141**, 967–76 (2017). 10.1002/ijc.30796 PMC5518206 28542846

[28] Smeby J, Sveen A, Merok MA, Danielsen SA, Eilertsen IA, Guren MG, et al. CMS-dependent prognostic impact of KRAS and BRAFV600E mutations in primary colorectal cancer. *Ann Oncol* **29**, 1227–34 (2018). 10.1093/annonc/mdy085 PMC5961317 29518181

[29] Smeby J, Sveen A, Bergsland CH, Eilertsen IA, Danielsen SA, Eide PW, et al. Exploratory analyses of consensus molecular subtype-dependent associations of TP53 mutations with immunomodulation and prognosis in colorectal cancer. *ESMO Open* **4**, e000523 (2019). 10.1136/esmoopen-2019-000523 PMC6598553 31321083

[30] Glaire MA, Domingo E, Sveen A, Bruun J, Nesbakken A, Nicholson G, et al. Tumour-infiltrating CD8+ lymphocytes and colorectal cancer recurrence by tumour and nodal stage. *Br J Cancer* **121**, 474–82 (2019). 10.1038/s41416-019-0540-4 PMC6738075 31388185

[31] Sveen A, Bruun J, Eide PW, Eilertsen IA, Ramirez L, Murumagi A, et al. Colorectal cancer consensus molecular subtypes translated to preclinical models uncover potentially targetable cancer cell dependencies. *Clin Cancer Res* **24**, 794–806 (2018). 10.1158/1078-0432.CCR-17-1234 29242316

[32] Irizarry RA, Hobbs B, Collin F, Beazer-Barclay YD, Antonellis KJ, Scherf U, et al. Exploration, normalization, and summaries of high density oligonucleotide array probe level data. *Biostatistics* **4**, 249–64 (2003). 10.1093/biostatistics/4.2.249 12925520

[33] Gautier L, Cope L, Bolstad BM, Irizarry RA. affy-analysis of Affymetrix GeneChip data at the probe level. *Bioinformatics* **20**, 307–15 (2004). 10.1093/bioinformatics/btg405 14960456

[34] Dai M, Wang P, Boyd AD, Kostov G, Athey B, Jones EG, et al. Evolving gene/transcript definitions significantly alter the interpretation of GeneChip data. *Nucleic Acids Res* **33**, e175 (2005). 10.1093/nar/gni179 PMC1283542 16284200

[35] Leek JT, Johnson WE, Parker HS, Jaffe AE, Storey JD. The sva package for removing batch effects and other unwanted variation in high-throughput experiments. *Bioinformatics* **28**, 882–3 (2012). 10.1093/bioinformatics/bts034 PMC3307112 22257669

[36] Huber W, Carey VJ, Gentleman R, Anders S, Carlson M, Carvalho BS, et al. Orchestrating high-throughput genomic analysis with Bioconductor. *Nat Methods* **12**, 115–21 (2015). 10.1038/nmeth.3252 PMC4509590 25633503

[37] Danaher P, Warren S, Dennis L, D’Amico L, White A, Disis ML, et al. Gene expression markers of Tumor Infiltrating Leukocytes. *J Immunother Cancer* **5**, 18 (2017). 10.1186/s40425-017-0215-8 PMC5319024 28239471

[38] Newman AM, Steen CB, Liu CL, Gentles AJ, Chaudhuri AA, Scherer F, et al. Determining cell type abundance and expression from bulk tissues with digital cytometry. *Nat Biotechnol* **37**, 773–82 (2019). 10.1038/s41587-019-0114-2 PMC6610714 31061481

[39] Miao YR, Zhang Q, Lei Q, Luo M, Xie GY, Wang H, et al. ImmuCellAI: a unique method for comprehensive T-cell subsets abundance prediction and its application in cancer immunotherapy. *Adv Sci (Weinh)* **7**, 1902880 (2020). 10.1002/advs.201902880 PMC7141005 32274301

[40] Enfield KSS, Martin SD, Marshall EA, Kung SHY, Gallagher P, Milne K, et al. Hyperspectral cell sociology reveals spatial tumor-immune cell interactions associated with lung cancer recurrence. *J Immunother Cancer* **7**, 13 (2019). 10.1186/s40425-018-0488-6 PMC6335759 30651131

[41] Punt CJ, Buyse M, Kohne CH, Hohenberger P, Labianca R, Schmoll HJ, et al. Endpoints in adjuvant treatment trials: a systematic review of the literature in colon cancer and proposed definitions for future trials. *J Natl Cancer Inst* **99**, 998–1003 (2007). 10.1093/jnci/djm024 17596575

[42] Sauerbrei W, Schumacher M. A bootstrap resampling procedure for model building: application to the Cox regression model. *Stat Med* **11**, 2093–109 (1992). 10.1002/sim.4780111607 1293671

[43] Yaqub S, Henjum K, Mahic M, Jahnsen FL, Aandahl EM, Bjørnbeth BA, et al. Regulatory T cells in colorectal cancer patients suppress anti-tumor immune activity in a COX-2 dependent manner. *Cancer Immunol Immunother* **57**, 813–21 (2008). 10.1007/s00262-007-0417-x PMC11030670 17962941

[44] Salama P, Phillips M, Grieu F, Morris M, Zeps N, Joseph D, et al. Tumor-infiltrating FOXP3+ T regulatory cells show strong prognostic significance in colorectal cancer. *J Clin Oncol* **27**, 186–92 (2009). 10.1200/JCO.2008.18.7229 19064967

[45] Frey DM, Droeser RA, Viehl CT, Zlobec I, Lugli A, Zingg U, et al. High frequency of tumor-infiltrating FOXP3(+) regulatory T cells predicts improved survival in mismatch repair-proficient colorectal cancer patients. *Int J Cancer* **126**, 2635–43 (2010). 10.1002/ijc.24989 19856313

[46] Pandiyan P, Zheng L, Ishihara S, Reed J, Lenardo MJ. CD4+CD25+Foxp3+ regulatory T cells induce cytokine deprivation–mediated apoptosis of effector CD4+ T cells. *Nat Immunol* **8**, 1353–62 (2007). 10.1038/ni1536 17982458

[47] Arce Vargas F, Furness AJS, Solomon I, Joshi K, Mekkaoui L, Lesko MH, et al. Fc-optimized anti-CD25 depletes tumor-infiltrating regulatory T cells and synergizes with PD-1 blockade to eradicate established tumors. *Immunity* **46**, 577–86 (2017). 10.1016/j.immuni.2017.03.013 PMC5437702 28410988

[48] Solomon I, Amann M, Goubier A, Arce Vargas F, Zervas D, Qing C, et al. CD25-Treg-depleting antibodies preserving IL-2 signaling on effector T cells enhance effector activation and antitumor immunity. *Nat Cancer* **1**, 1153–66 (2020). 10.1038/s43018-020-00133-0 PMC7116816 33644766

