# Supplementary material for: Spatial analysis and CD25-expression identify regulatory T cells as predictors of a poor prognosis in colorectal cancer
Source: Mod Pathol. 2022 Apr 28;35(9):1236–46. doi: 10.1038/s41379-022-01086-8 (PMC9424114; doi:10.1038/s41379-022-01086-8)
Supplement: Supplementary file 1 — Supplementary material [file 41379_2022_1086_MOESM1_ESM.docx]

**Supplementary Material**

Contents

[Supplementary figure 1 4](#_Toc99008269)

[Supplementary figure 2 5](#_Toc99008270)

[Supplementary figure 3 7](#_Toc99008271)

[Supplementary figure 4 8](#_Toc99008272)

[Supplementary figure 5 9](#_Toc99008273)

[Supplementary figure 6 10](#_Toc99008274)

[Supplementary figure 7 11](#_Toc99008275)

[Supplementary figure 8 12](#_Toc99008276)

[Supplementary figure 9 13](#_Toc99008277)

[Supplementary figure 10 14](#_Toc99008278)

[Supplementary figure 11 15](#_Toc99008279)

[Supplementary figure 12 16](#_Toc99008280)

[Supplementary figure 13 17](#_Toc99008281)

[Supplementary figure 14 18](#_Toc99008282)

[Supplementary figure 15 19](#_Toc99008283)

[Supplementary figure 16 20](#_Toc99008284)

[Supplementary figure 17 21](#_Toc99008285)

[Supplementary figure 18 22](#_Toc99008286)

[Supplementary figure 19 23](#_Toc99008287)

[Supplementary table 1 24](#_Toc99008288)

[Supplementary table 2 25](#_Toc99008289)

[Supplementary table 3 26](#_Toc99008290)

[Supplementary table 4 27](#_Toc99008291)

[Supplementary table 5 28](#_Toc99008292)

[Supplementary table 6 29](#_Toc99008293)

[Supplementary table 7 30](#_Toc99008294)

[Supplementary table 8 31](#_Toc99008295)

[Supplementary table 9 32](#_Toc99008296)

[Supplementary table 10 33](#_Toc99008297)

[Supplementary table 11 34](#_Toc99008298)

[Supplementary table 12 35](#_Toc99008299)

[Supplementary table 13 36](#_Toc99008300)

[Supplementary table 14 37](#_Toc99008301)

[Supplementary table 15 38](#_Toc99008302)

[Supplementary table 16 39](#_Toc99008303)

[Supplementary table 17 40](#_Toc99008304)

[Supplementary table 18 41](#_Toc99008305)

[Supplementary table 19 42](#_Toc99008306)

[Supplementary reference 43](#_Toc99008307)


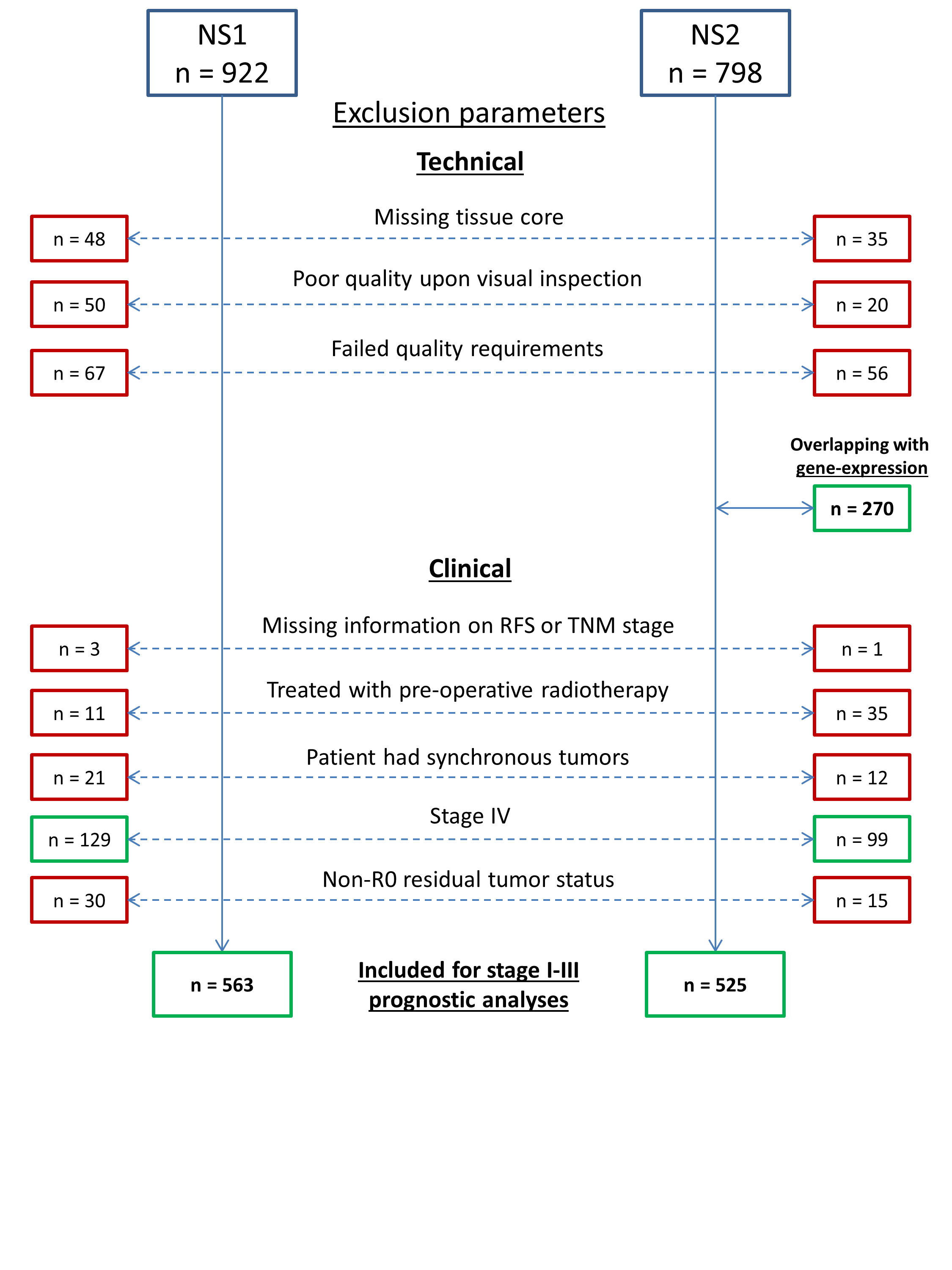


Supplementary figure 1**: Flowchart for exclusion of samples from the analyses.** After visual inspection and removal of destroyed tissue/necrotic tissue cores, a series of filters was applied to remove samples with inadequate amount of tissue or cells to include for analysis (specifically, the requirements were that the images of samples must have at least 5% tumor epithelial tissue, at least 100 tumor epithelial cells, at least 50 stromal cells and that there was at least an area of 150,000 pixels (0.0375mm^2^) tissue available for analysis). For prognostic evaluations, a set of filters based on clinical characteristics of the patients was applied, as indicated. Abbreviations: RFS; relapse-free survival, TNM; tumor-node-metastasis.


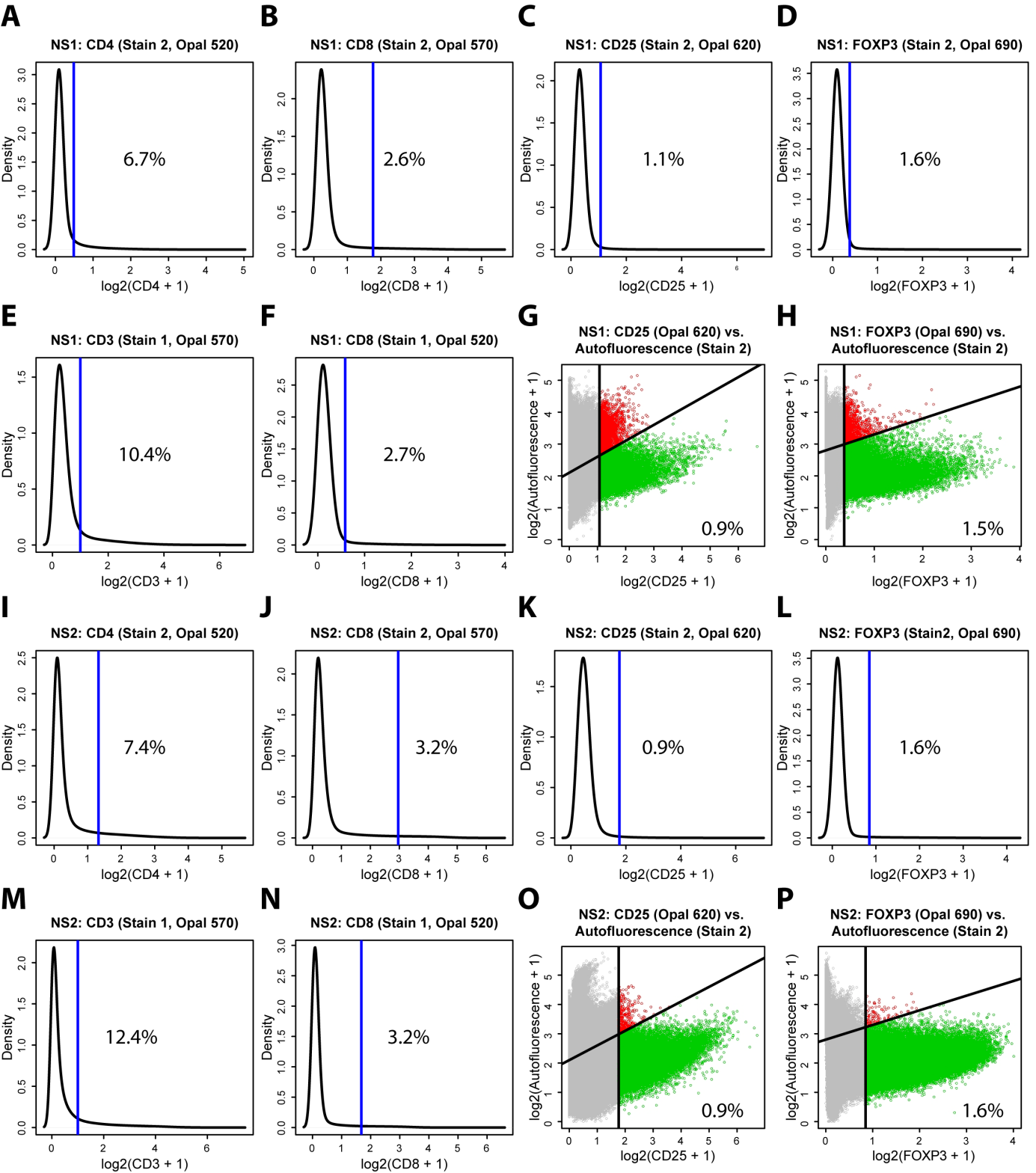


Supplementary figure 2**: Density plots and thresholds for marker positivity.** Density plots of mean fluorescence intensity values per cell were plotted for each marker within each of the two series. Density plots for Norwegian series 1 (NS1) are shown in figures A-F, while density plots for Norwegian series 2 (NS2) are shown in figures I-N. Additionally, we found some interference between autofluorescence and the Opal fluorophores 620 and 690. By manual inspection of images in inForm, we found that this was largely due to erythrocytes with very high autofluorescence levels falsely giving signal to neighboring cells. Therefore an additional filtering step was used to remove cells with a high autofluorescence to fluorophore intensity for these two fluorophores (NS1; figures G and H, NS2; figures O and P). Gray dots indicate cells excluded based on thresholds from density plot, red dots indicate cells excluded due to high autofluorescence and green cells indicate cells positive for the given marker. Percentages indicate the percentage of cells scored positive for the given marker within all cells of the series.


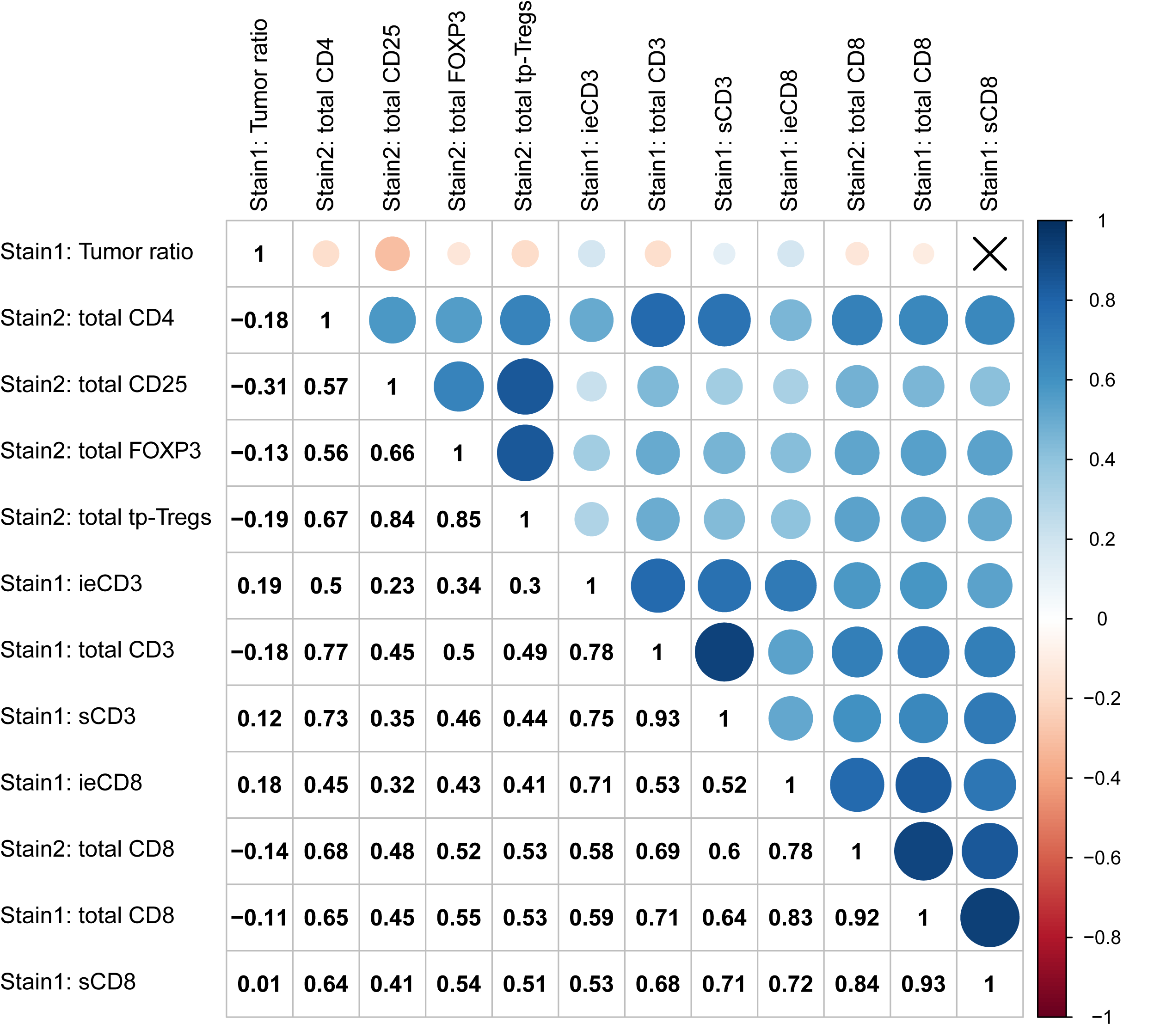


Supplementary figure 3**: Correlation-matrix for IHC-based immune cell infiltration-scores in stage I-III, R0-resected patients of the Norwegian series 2.** Stain 1 included epithelial (malignant cancer cell) markers and CD8^+^- and CD3^+^-cells were scored within the malignant (intraepithelial, ie) and stromal (s) compartment individually, as well as in the tissue as a whole (total). CD8^+^-cells were analyzed in both stains; the correlation was high (R = 0.92), indicating good agreement between the two tissue-sections. A tumor ratio measure was also calculated as the amount of epithelial cancer tissue divided by total tissue in the image; since immune cells are located to a larger extent in the stroma than epithelial cancer tissue, most immune-scores based on analysis of total tissue are negatively associated with an increased tumor ratio. All scores, except the tumor ratio, were log2-transformed and Pearson’s correlation metric was used to quantify associations between the variables. The order of variables was determined by hierarchical clustering of the correlation matrix. p-value above 0.05 is marked with a cross.


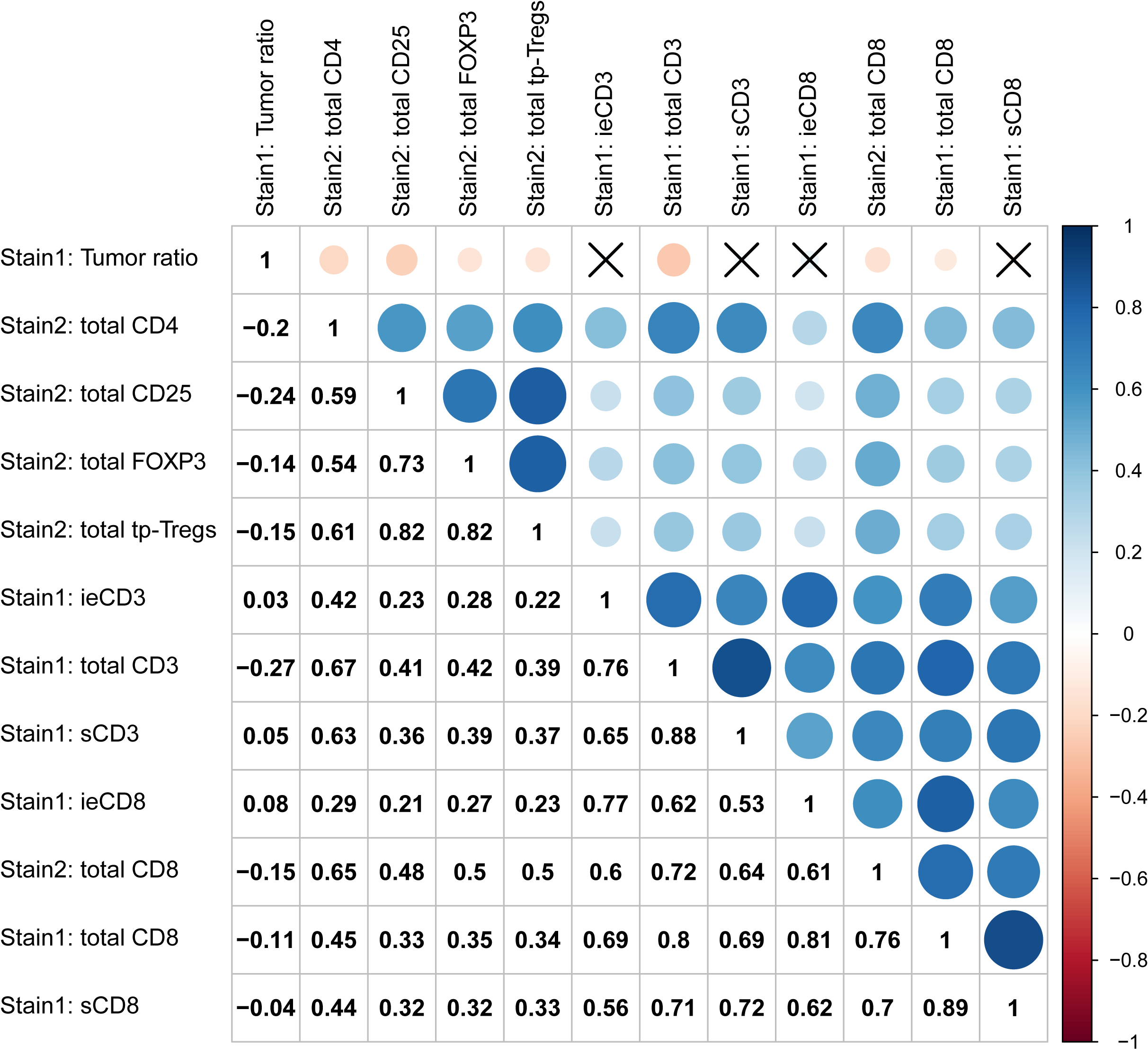


Supplementary figure 4**: Correlation-matrix for IHC-based immune cell infiltration-scores in stage I-III, R0-resected patients of the Norwegian series 1.** Stain 1 included epithelial (malignant cancer cell) markers and CD8^+^- and CD3^+^-cells were scored within the malignant (intraepithelial, ie) and stromal (s) compartment individually, as well as in the tissue as a whole (total). Overall, correlations between the various immune cell populations were similar to what was found in the Norwegian series 2 (Supplementary figure 3). In general, the R*-*values were somewhat lower, which is likely explained by the smaller sample-sizes used to construct the Norwegian series 1 compared to the Norwegian series 2 (0.6mm *vs* 1.0mm diameter cores). All scores, except the tumor ratio, were log2-transformed and Pearson’s correlation metric was used to quantify associations between the variables. The order of variables was set to match the order in Supplementary figure 3. p-values above 0.05 are marked with crosses.


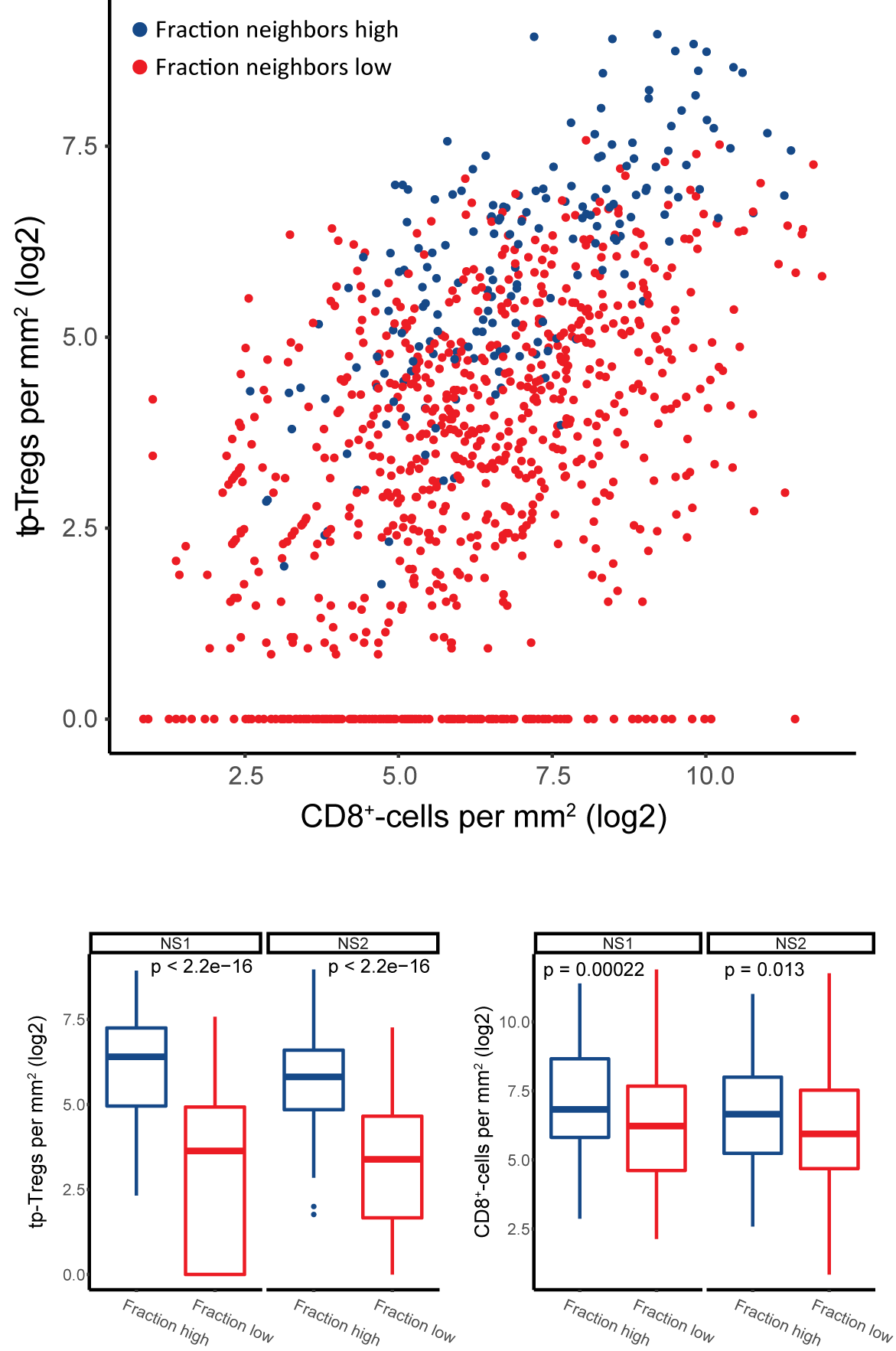


Supplementary figure 5**: Groups defined by spatial proximity without the use of Monte-Carlo permutations and significance testing show strong associations with absolute immune cell infiltration.**The fraction of CD8^+^-cells neighboring at least one triple-positive Treg (tp-Treg) was calculated and samples of the pooled Norwegian series were grouped such that the same number of samples were classified as having a high fraction of interacting cells, as the number of samples that were classified as having significant spatial interactions (18% of the total samples, compare to Figure 3D). High fractions of CD8^+^-cells neighboring triple-positive Tregs was highly associated with absolute triple-positive Treg infiltration, and also associated to absolute CD8^+^-cell infiltration. Abbreviations: NS1; Norwegian series 1, NS2; Norwegian series 2.


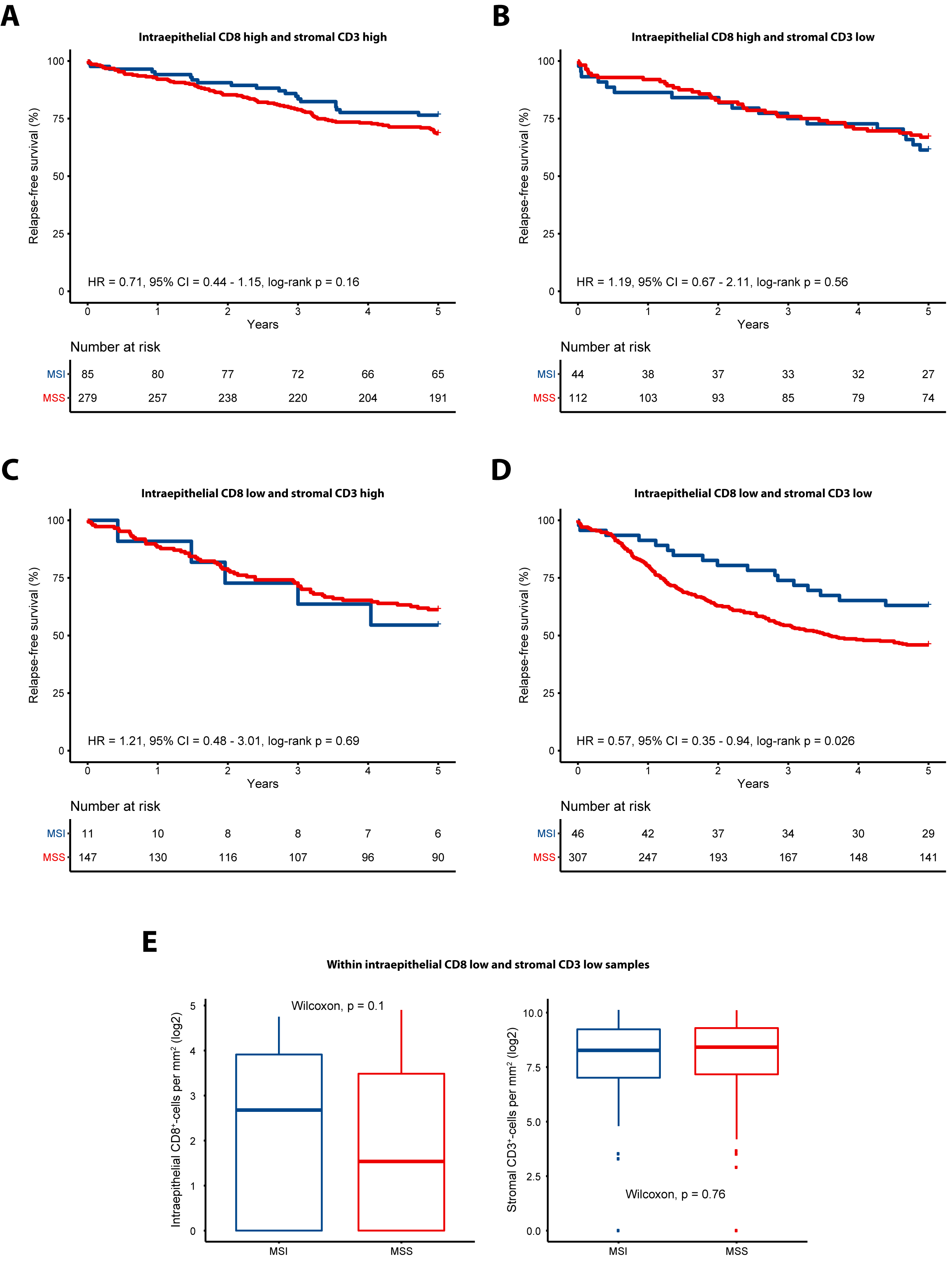


Supplementary figure 6**: Survival according to microsatellite instability status within patient subgroups defined by intraepithelial CD8 and stromal CD3 scores.** Survival according to MSI status was tested within the four groups defined by intraepithelial CD8 and stromal CD3 scores (A-D). No significant differences in cell densities were found between MSI and MSS samples within the intraepithelial CD8 low and stromal CD3 low subgroup (E).

**
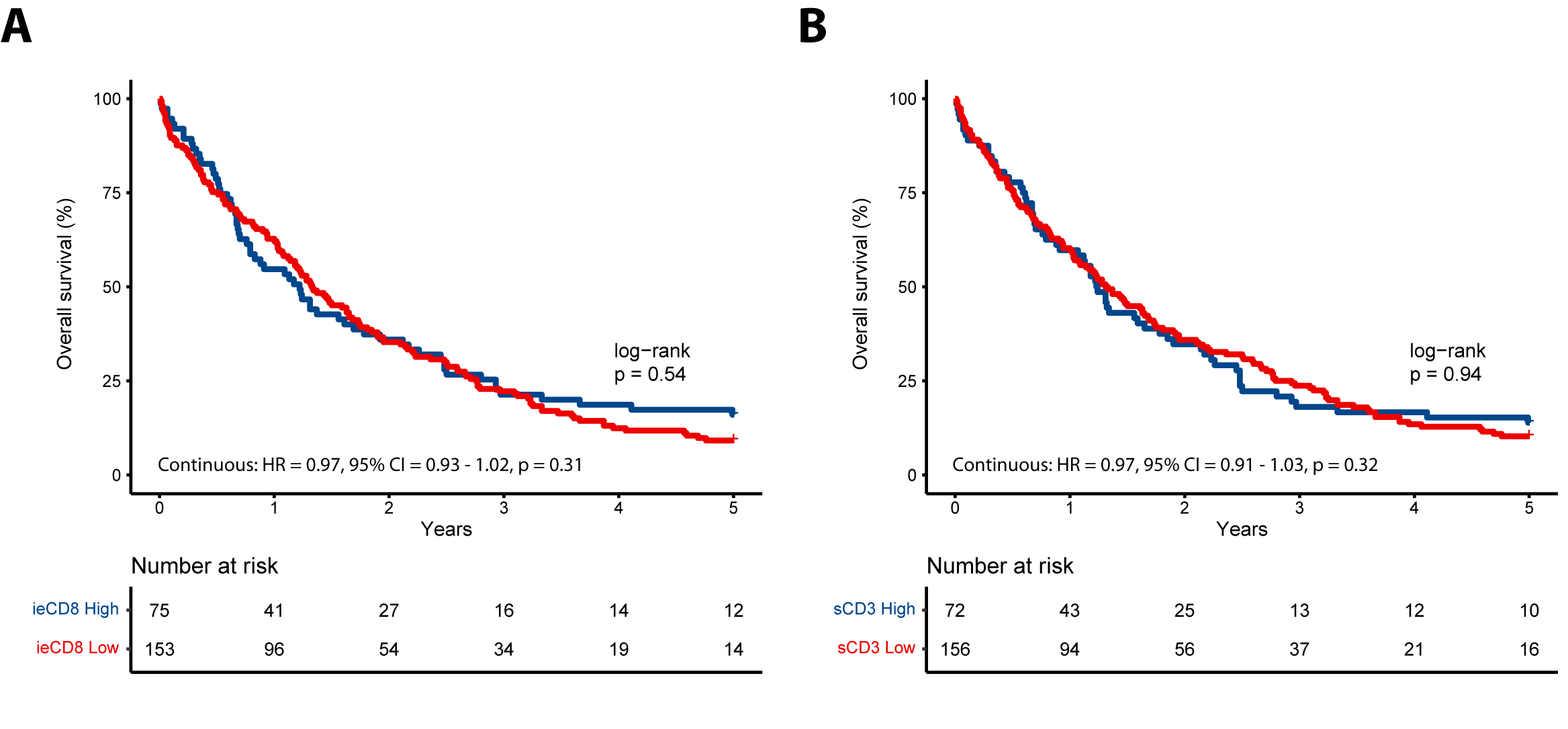
**

Supplementary figure 7**: Overall survival according to intraepithelial (ie) CD8 scores (A) and stromal (s) CD3 scores (B) in stage IV CRC patients.**


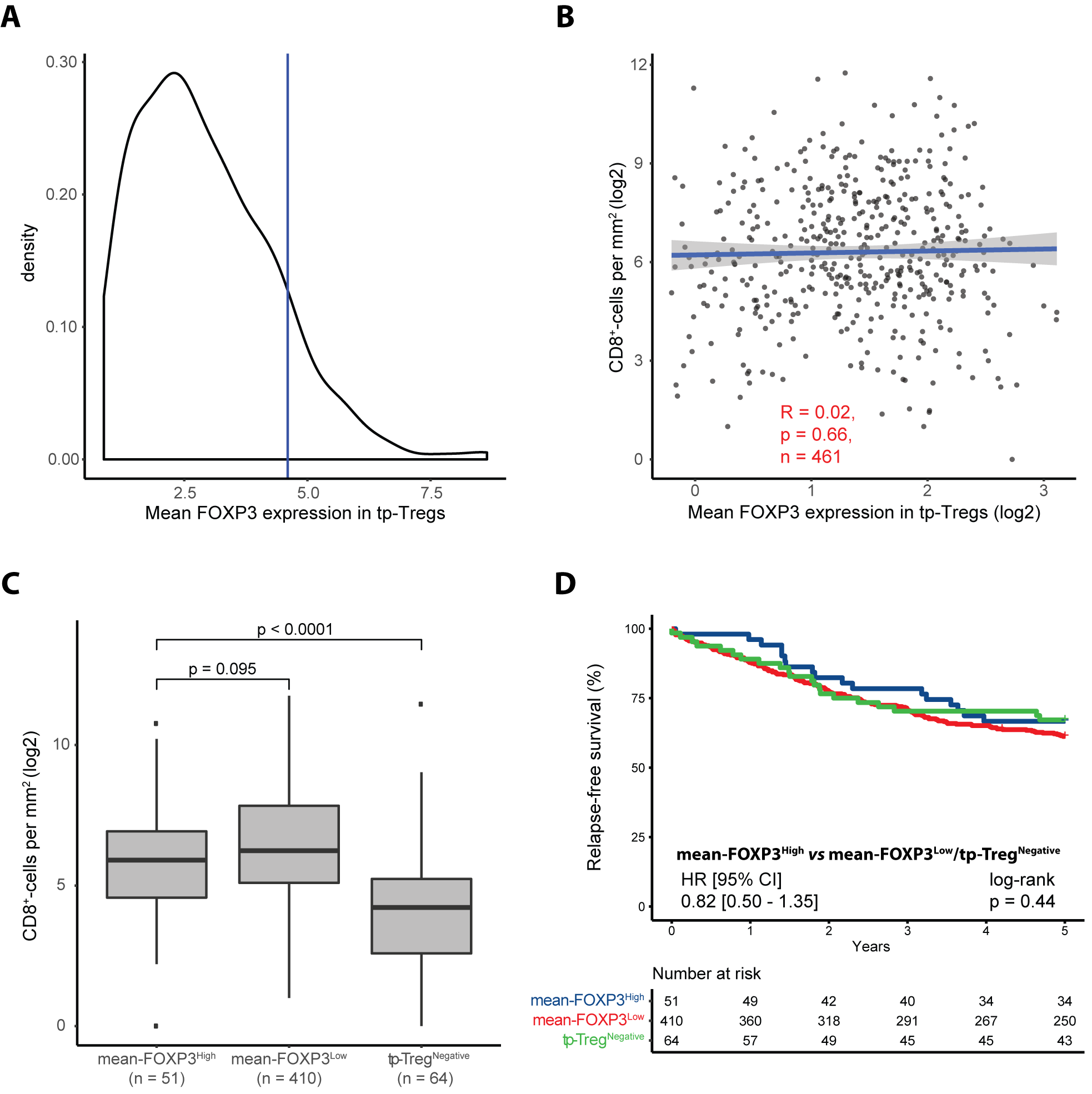


Supplementary figure 8**: Mean FOXP3 expression in triple-positive Tregs is not associated with prognosis in the Norwegian series 2.** Density-plot with the cutoff (blue vertical line) set for separating samples with low- and high-FOXP3-expressing triple-positive Tregs (A). The cutoff for FOXP3 was set according to the value that maximally separated groups in Kaplan-Meier survival analysis, since there was no obvious place in the curve to set a threshold for this variable. There was no significant association between mean FOXP3-expression in triple-positive Tregs and CD8^+^-infiltration scores (B) (samples with no detected triple-positive Tregs are excluded from the plot). Boxplot according to mean-FOXP3 expression following dichotomization (C). Samples with no detected triple-positive Tregs are included as a separate category (tp-Treg^Negative^). Kaplan-Meier survival curves according to the three groups defined by mean-FOXP3 expression in triple-positive Tregs (D). Patients with mean-FOXP3^High^ samples were compared to those with mean-FOXP3^Low^ and those with no triple-positive Tregs (tp-Treg^Negative^) combined.

**
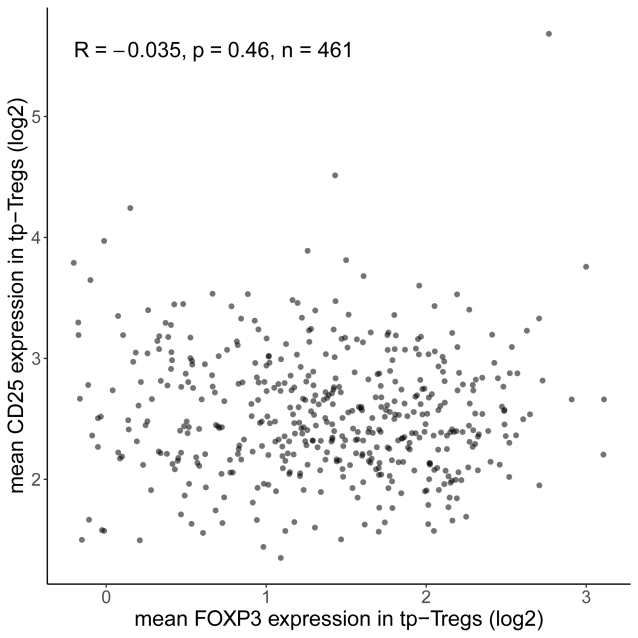
**

Supplementary figure 9**: Mean FOXP3 expression plotted against mean CD25 expression within individual samples’ triple-positive Tregs (tp-Tregs) in the Norwegian series 2.**


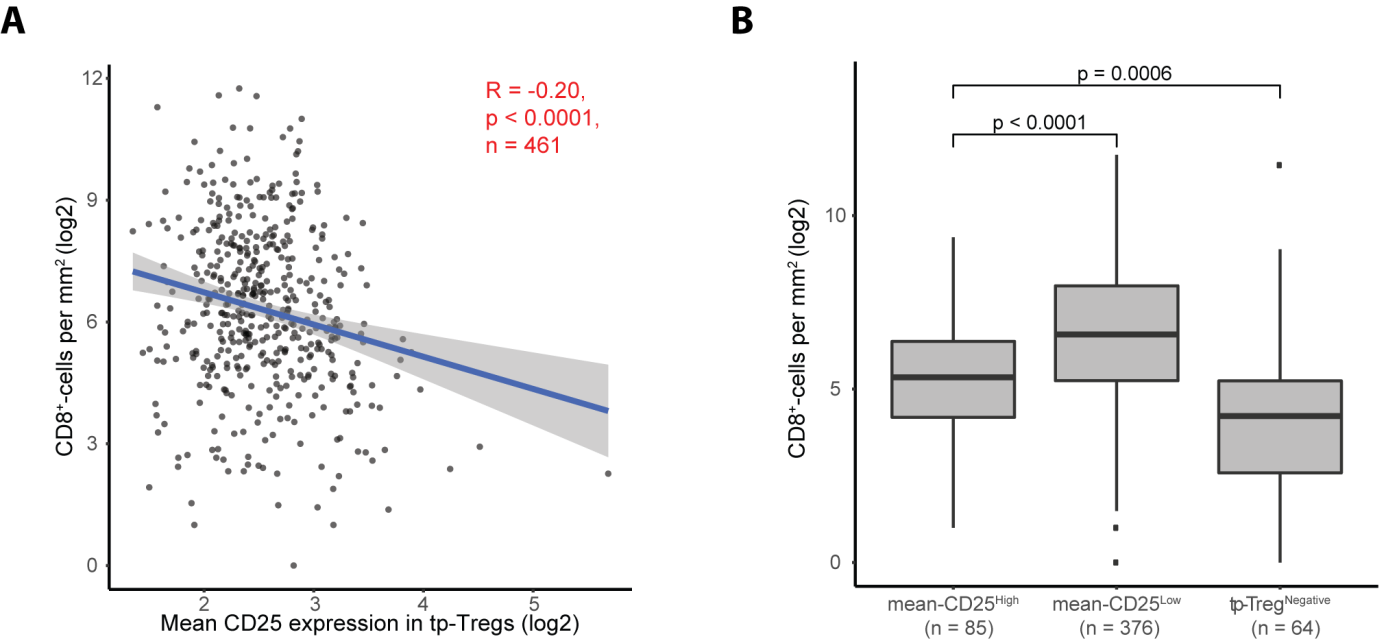


Supplementary figure 10**: Mean CD25 expression in triple-positive Tregs is associated with low infiltration of CD8+-cells in the Norwegian series 2.** Mean CD25-expression in triple-positive Tregs plotted against CD8^+^-infiltration scores shows a negative correlation between these variables (A) (samples with no detected triple-positive Tregs are excluded from the plot). Boxplot according to CD25-expression following dichotomization (B). Samples with no detected triple-positive Tregs are included as a separate category (tp-Treg^Negative^).


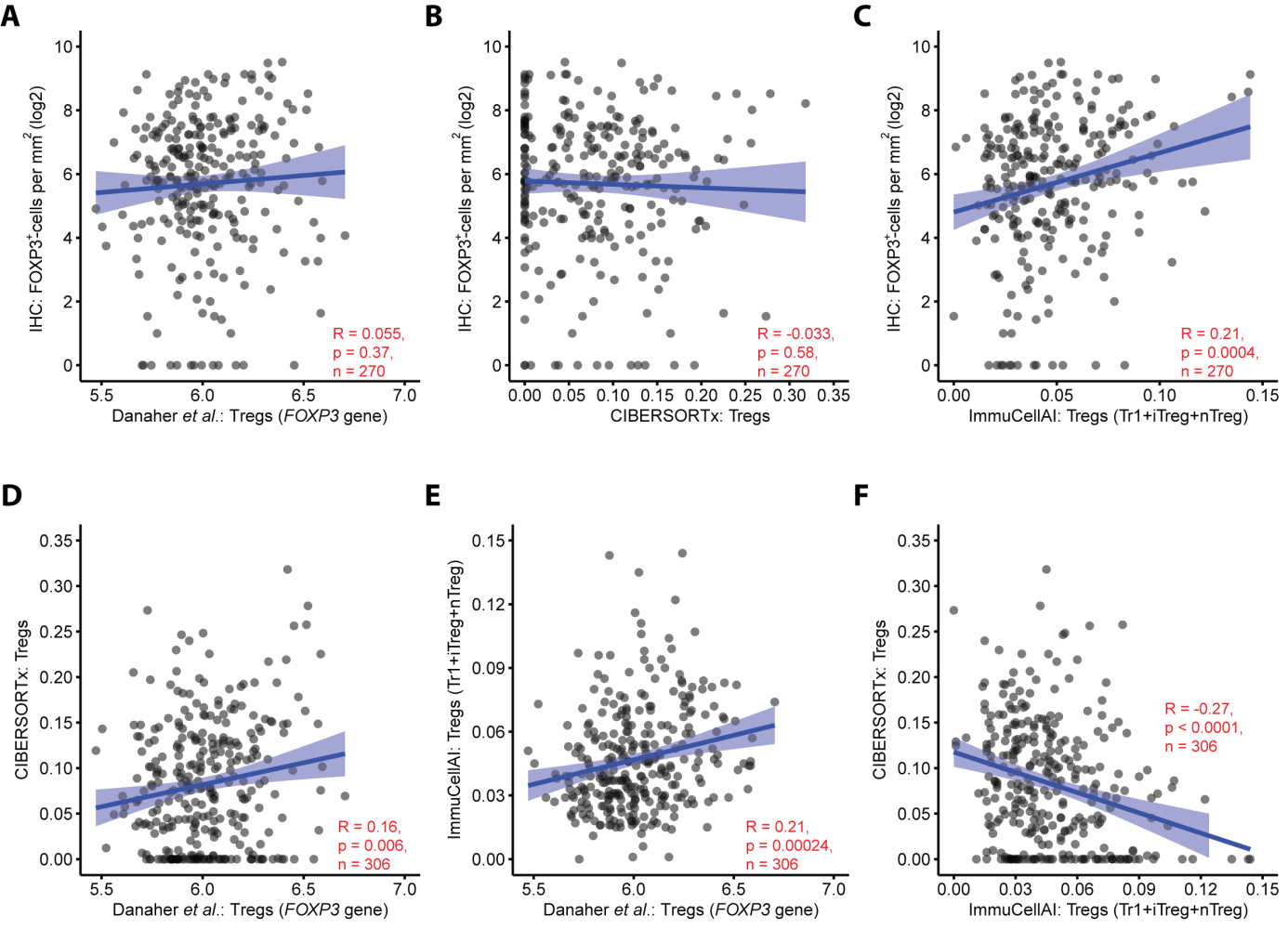


Supplementary figure 11**: Correlations between gene-expression- and IHC-based scoring of Tregs.**
Treg-infiltration scored by the algorithm published by Danaher *et* *al*. **(A)**, CIBERSORTx **(B)** and ImmuCellAI **(C)** were compared to FOXP3^+^-cell infiltration by IHC. The Treg-score provided by Danaher *et al*. is equal to the *FOXP3*-gene-expression score in the sample. The ImmuCellAI algorithm scores three populations of T-regulatory cells, Tr1, iTreg and nTreg. Comparisons between IHC-based scoring and ImmuCellAI were therefore tested for each of these scores individually, and also upon summing the three. The sum of the three scores provided the best correlation with IHC. The three gene-expression based scores were also compared to each other **(D-F)**. As a note, with respect to comparisons with the ImmuCellAI scores, Danaher *et al*.’s Treg-score correlated the best with Tr1-cells (R = 0.32, p < 0.0001, plot not included) and CIBERSORTx’s Treg-score correlated the best with iTreg (R = 0.17, p = 0.0029, plot not included).

**
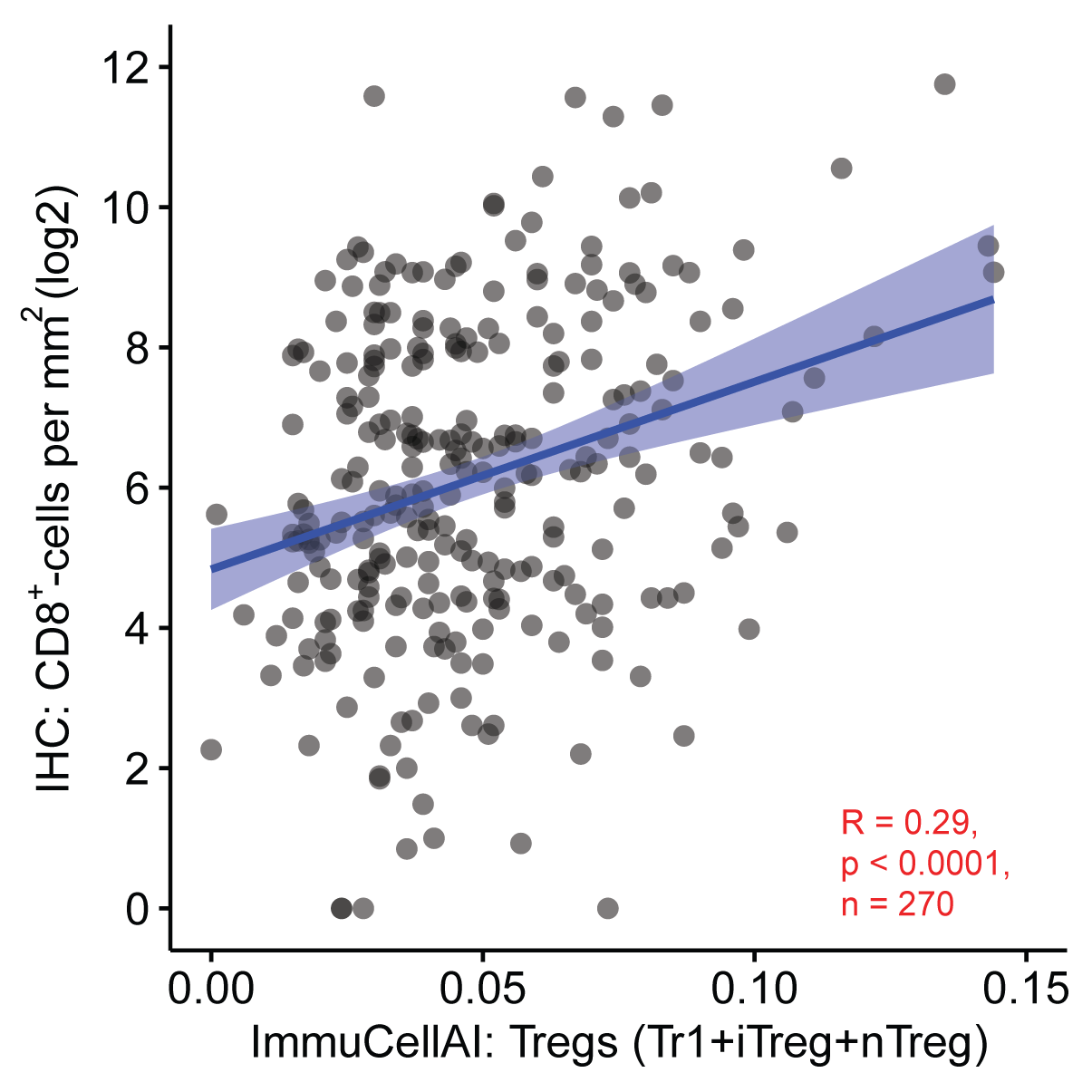
**

Supplementary figure 12**: Association between ImmuCellAI-scores for Tregs and CD8+-cell infiltration scored by IHC.** The Treg score for ImmuCellAI was obtained by summing the three individual scores for Tregs output by the algorithm.


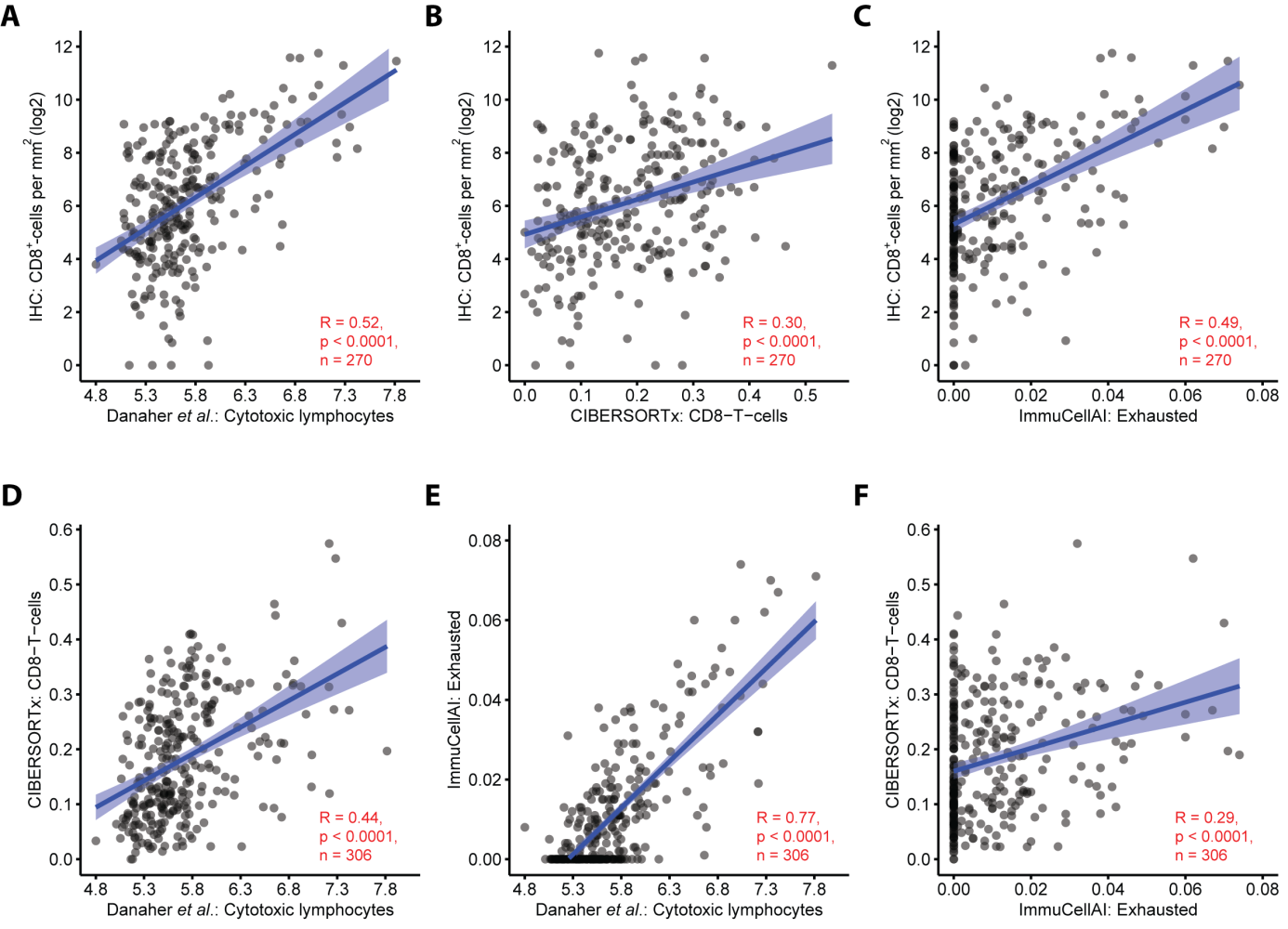


Supplementary figure 13**: Correlations between gene-expression- and IHC-based scoring of CD8-populations.** CD8^+^-infiltration scored by IHC was compared to scores provided by the algorithm published by Danaher *et al*. **(A)**, CIBERSORTx **(B)** and ImmuCellAI **(C)**. CD8^+^-cell infiltration scored by IHC was also tested against the CD8-T-cell score provided by the algorithm by Danaher *et al*.; the correlation was weaker (R = 0.39, p < 0.0001) and only the plot against cytotoxic lymphocytes is included. With respect to the ImmuCellAI algorithm, CD8^+^-cell infiltration scored by IHC was also tested against the scores provided for CD8 T (R = 0.16, p = 0.01), CD8 naïve (R = -0.24, p < 0.0001) and cytotoxic T cells (R = 0.25, p < 0.0001). The three gene-expression based scores were also compared to each other **(D-F)**. Although the cytotoxic lymphocyte score from Danaher’s algorithm correlated the best with the IHC-based score of CD8^+^-cells, the CD8-T-cell score from Danaher’s algorithm correlated better with the CD8-T-cell score from CIBERSORTx (R = 0.56, p < 0.0001, plot not included). All comparisons were performed between the three other CD8-cell populations scored by ImmuCellAI (described above) and the cytotoxic lymphocyte and CD8-T-cell scores from Danaher’s algorithm; the highest correlation was found for the populations shown in E, in line with what was found for the comparisons with the IHC-data. All comparisons were also performed between the three other CD8-cell populations scored by ImmuCellAI (described above) and the CD8-T-cells scored by CIBERSORTx; again, the highest correlation was found for the populations shown in F, which were the same populations found to correlate best with IHC.


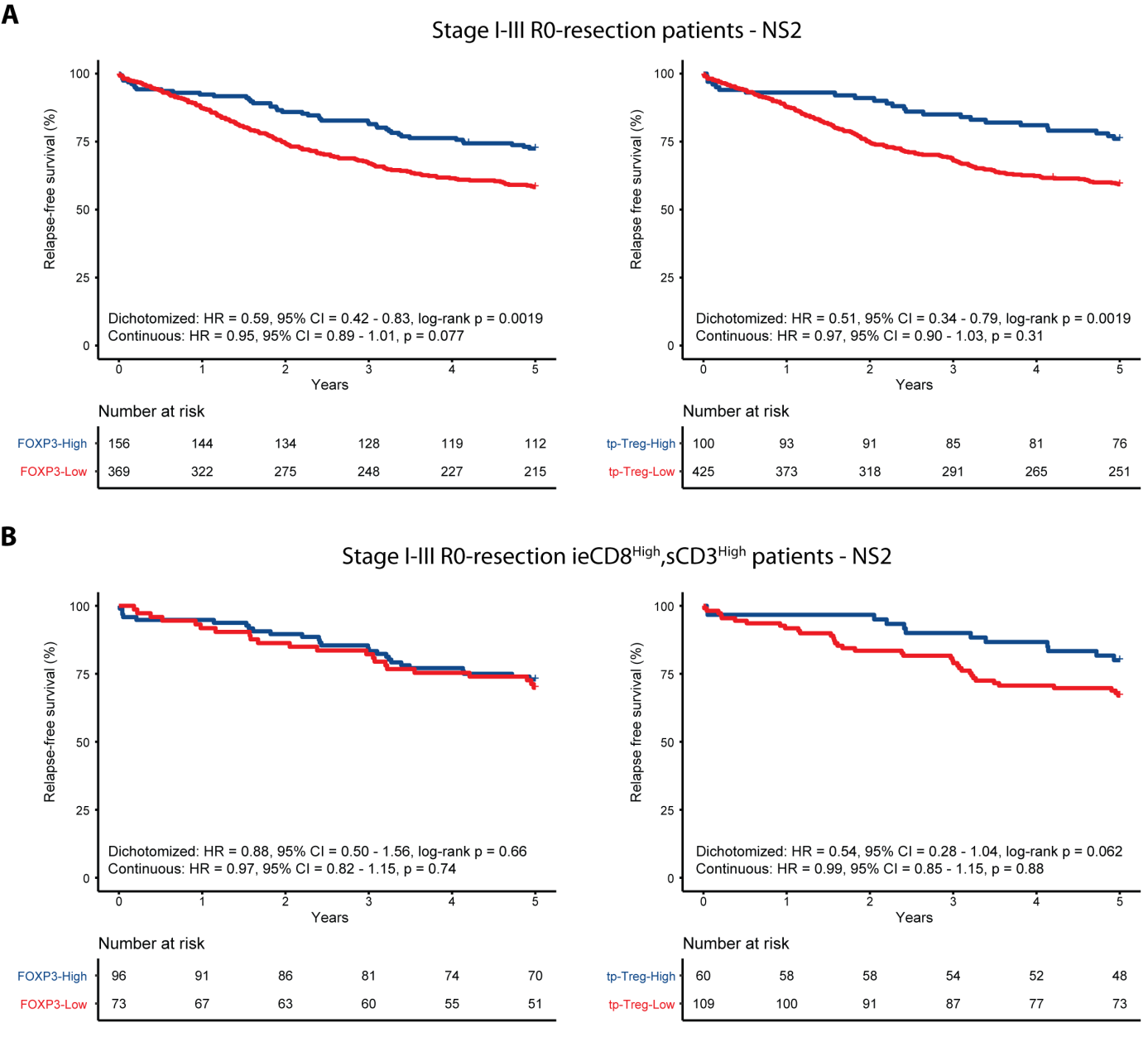


Supplementary figure 14**: Prognostic value of FOXP3 (left) and triple-positive Treg (right) infiltration in the Norwegian series 2.** Prognostic value was determined within stage I-III R0 patients of the Norwegian series 2 (A), and within the subset of patients with concomitant high intraepithelial CD8 and high stromal CD3 scores (B). Cutoffs for dichotomization into high/low infiltration of FOXP3/triple-positive Tregs were determined within all stage I-III R0 patients and set at the values that maximized the differences in survival curves. Analyses using log2-transformed continuous scores are also presented. Abbreviations: ieCD8; intraepithelial CD8, sCD3; stromal CD3, NS2; Norwegian series 2.


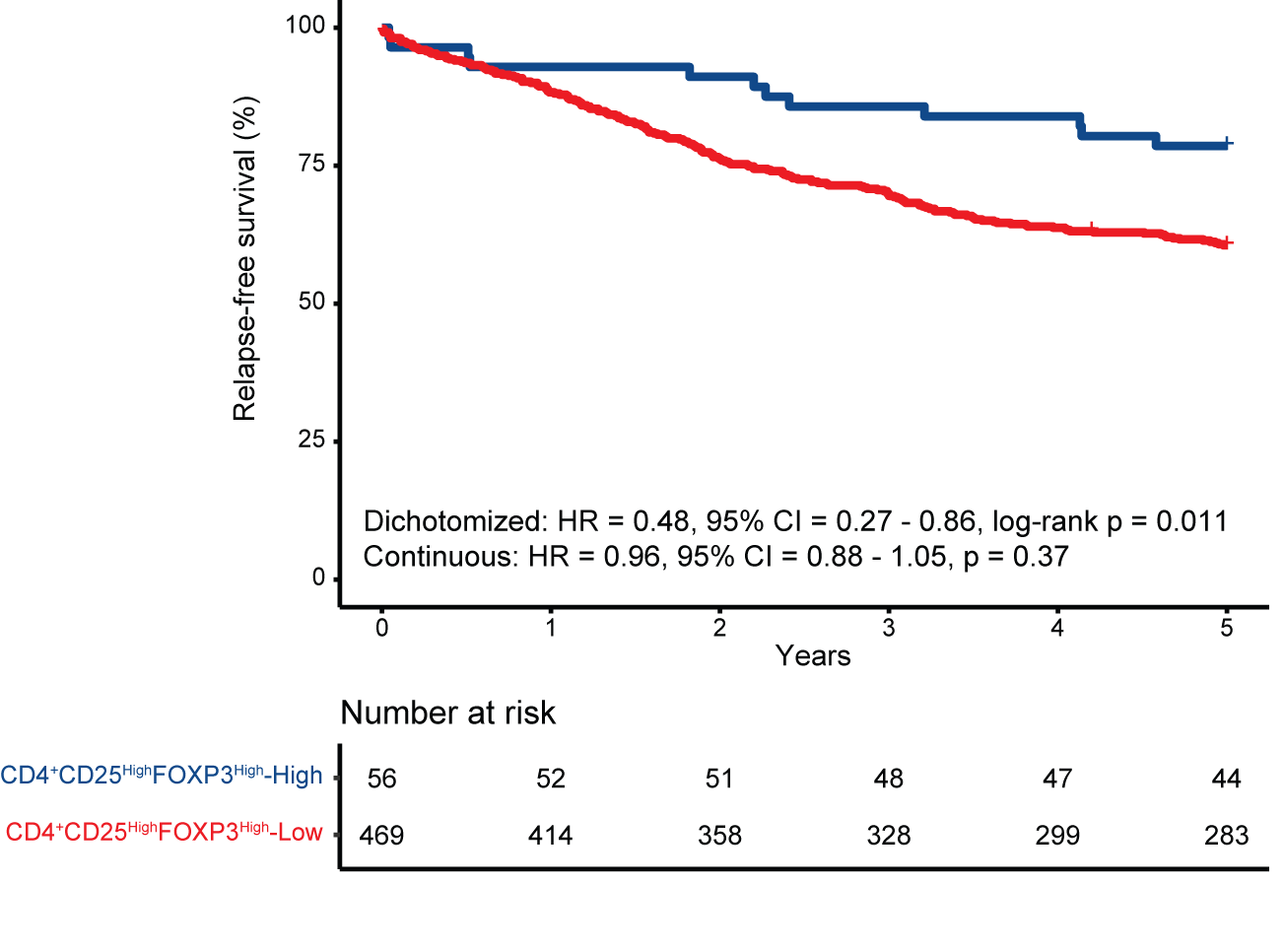


Supplementary figure 15**: Prognostic value of infiltrating triple-positive Tregs with high levels of CD25 and FOXP3 (CD4+CD25HighFOXP3High) in the Norwegian series 2.** Cutoff for dichotomization into high/low infiltration was set at the value that maximized the difference in survival curves. Analysis using the log2-transformed continuous score is also presented.


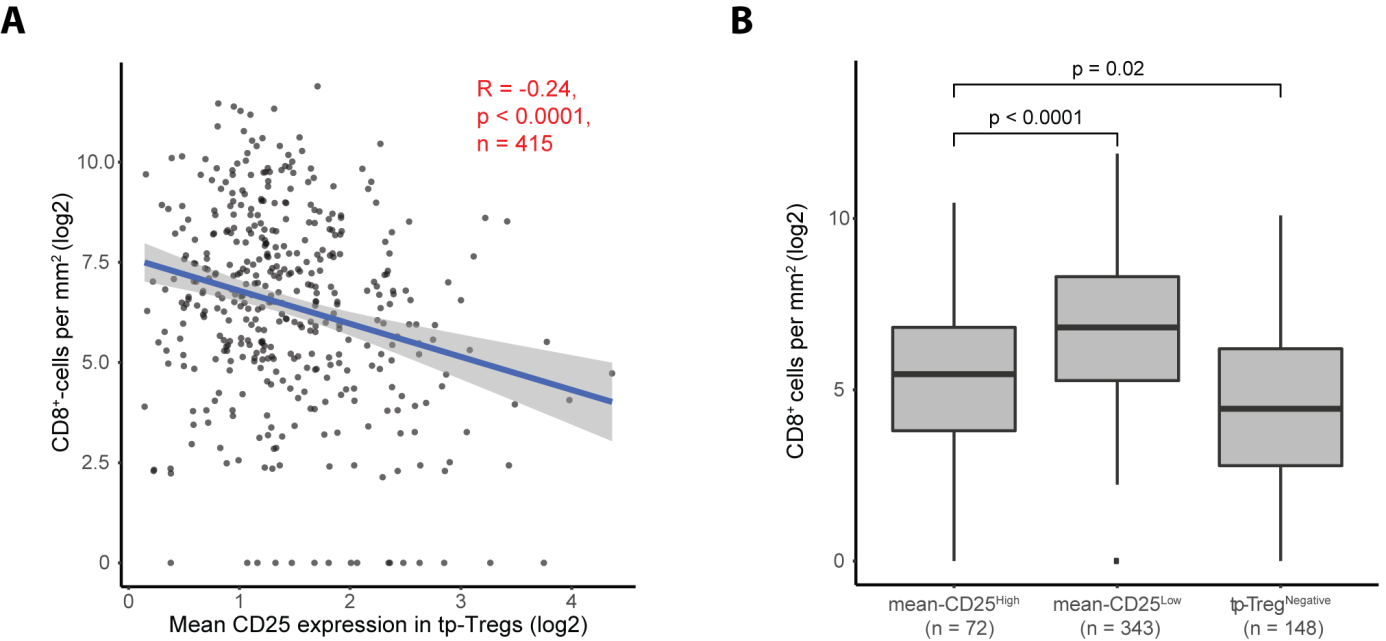


Supplementary figure 16**: Mean CD25 expression in triple-positive Tregs is associated with low infiltration of CD8+-cells in the Norwegian series 1.** Mean CD25 expression in triple-positive Tregs plotted against CD8^+^-infiltration scores shows a negative correlation between these variables (A) (samples with no detected triple-positive Tregs are excluded from the plot). Boxplot according to CD25 expression following dichotomization (B). Samples with no detected triple-positive Tregs are included as a separate category (tp-Treg^Negative^).


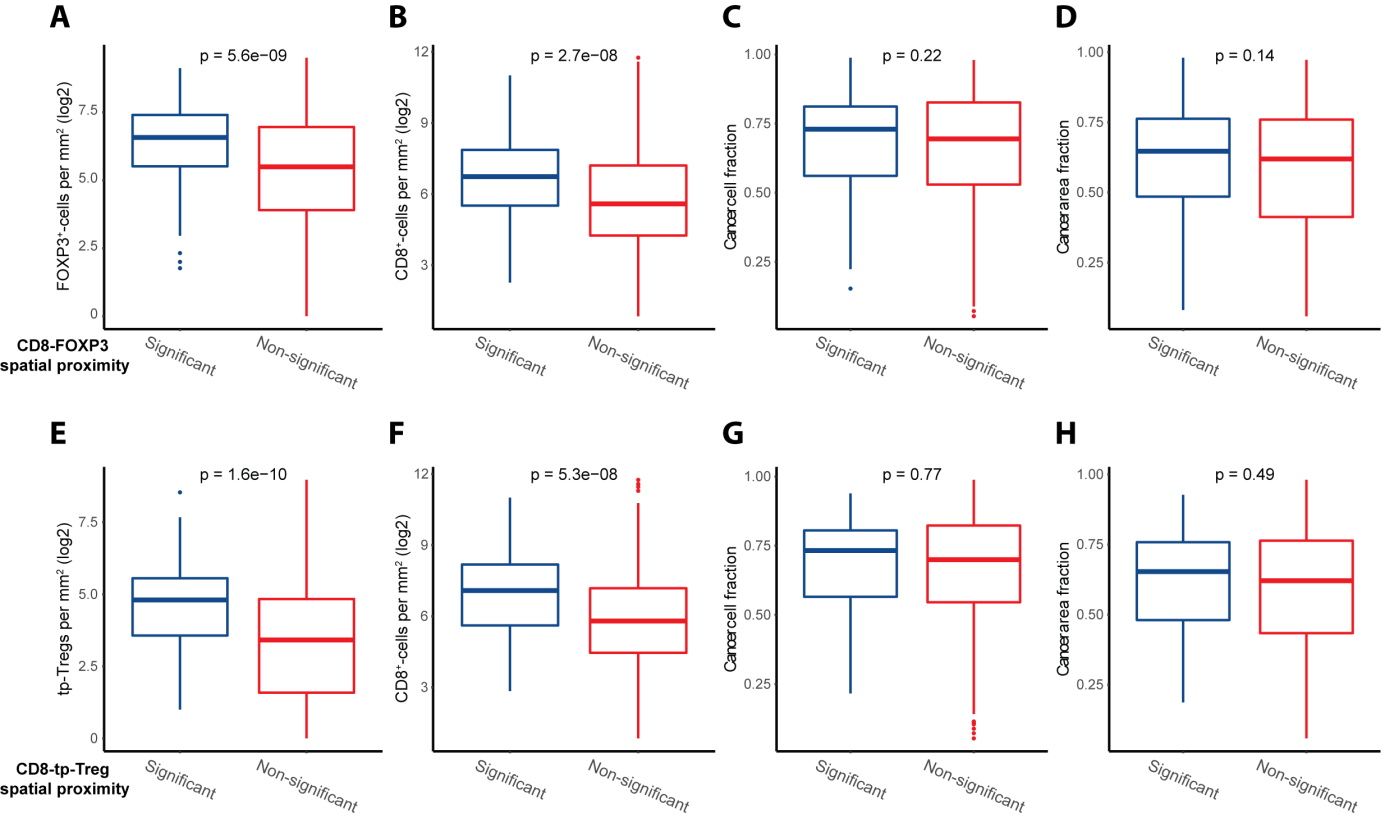


Supplementary figure 17**: Associations between the spatial proximity groups and absolute immune infiltration scores and cancer cell/area fractions.**Samples of the Norwegian series 2 were split according to whether they were determined to have significant or non-significant associations between neighboring CD8^+^-cells and FOXP3^+^-cells (A-D) or triple-positive Tregs (E-F).


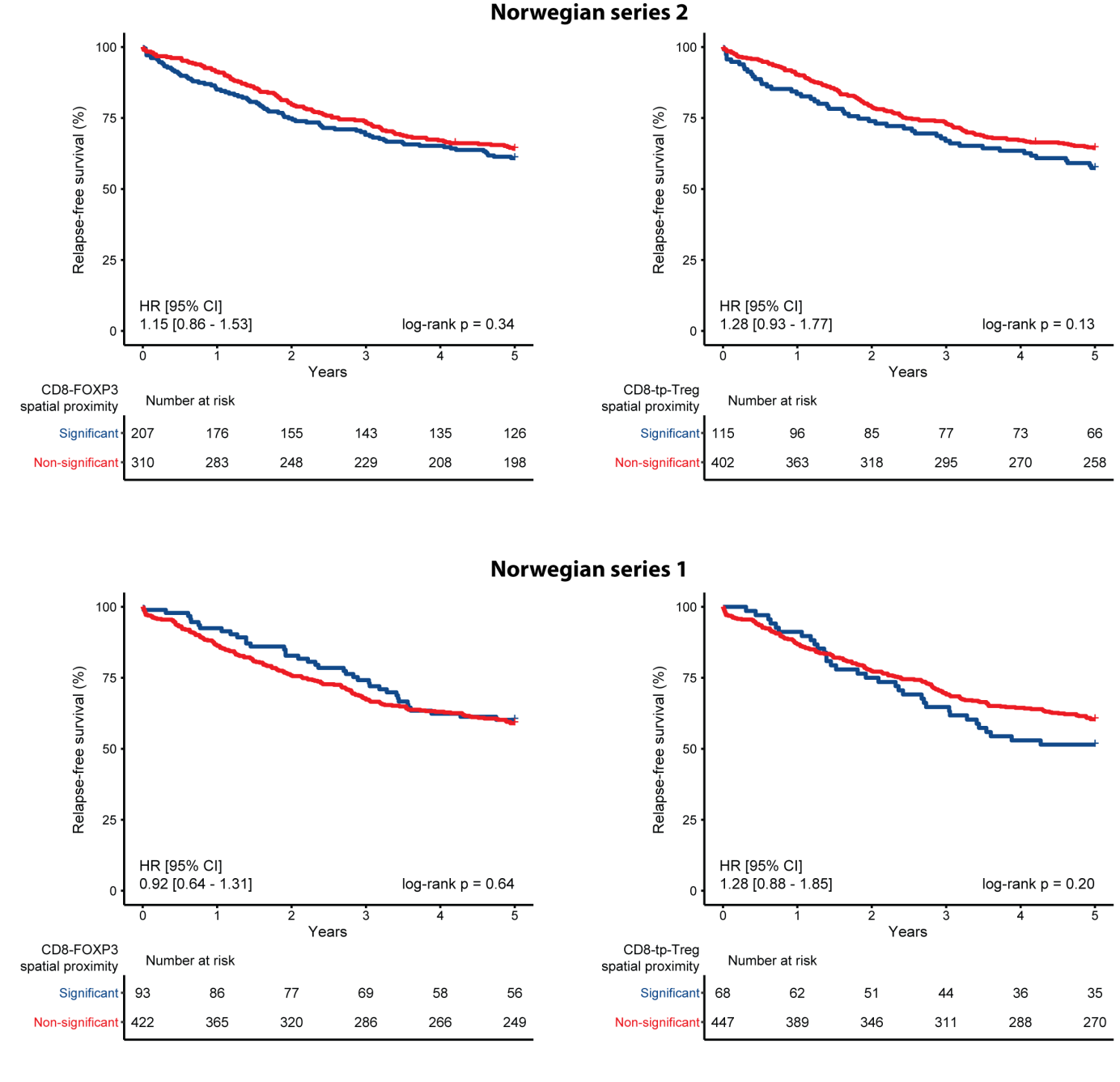


Supplementary figure 18**: Kaplan-Meier survival analysis based on spatial relationships between CD8+-cells and Tregs.** Analysis was performed within the Norwegian series 2 (top) and the Norwegian series 1 (bottom) according to significant/non-significant spatial relationships between CD8^+^- and FOXP3^+^-cells (left) and CD8^+^-cells and triple-positive Tregs (right).


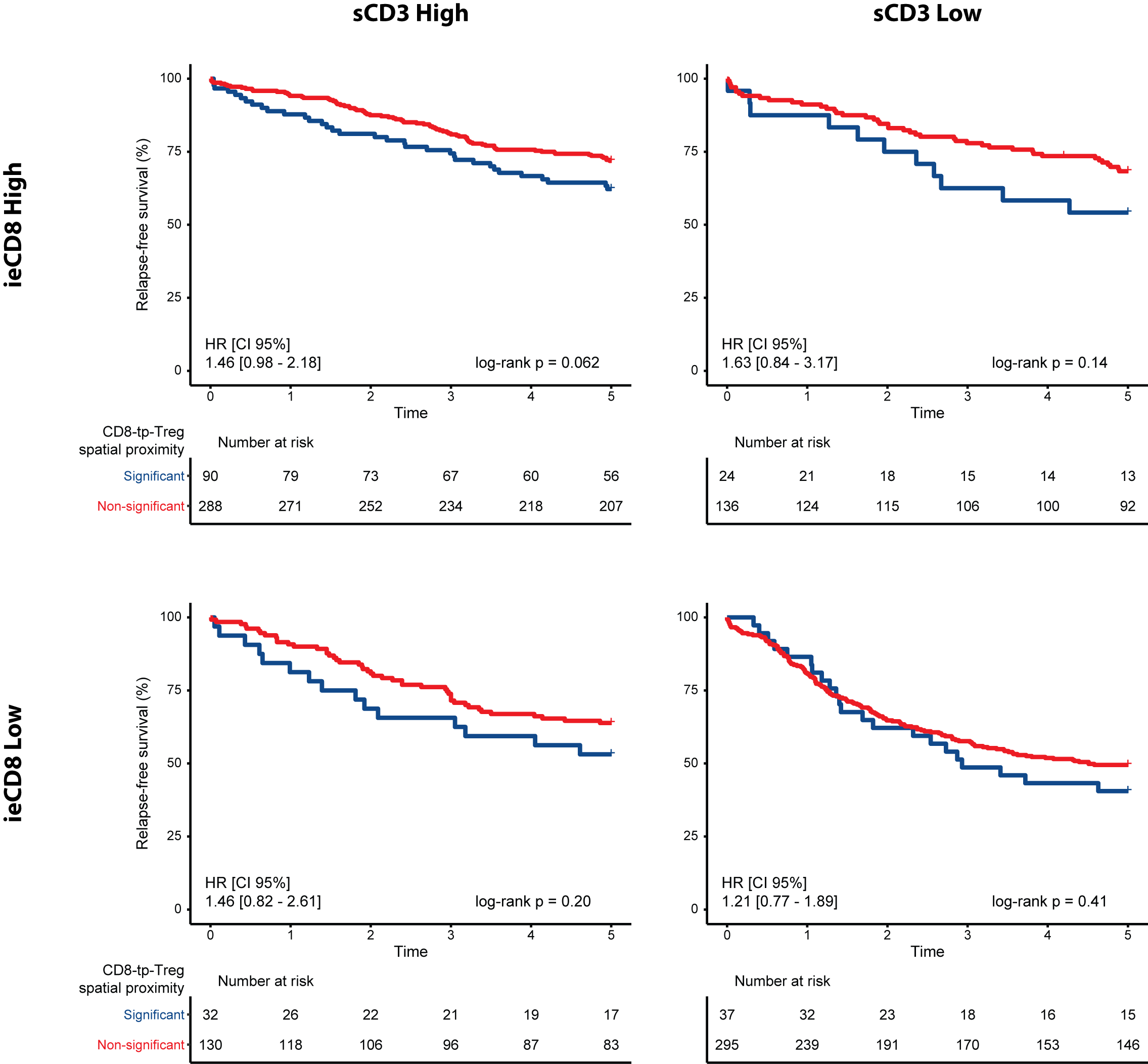


Supplementary figure 19**: Survival according to CD8-to-triple-positive Treg spatial groups, split according to intraepithelial (ie) CD8 and stromal (s) CD3 infiltration.** Analyses were performed upon pooling the two series.

Supplementary table 1**: REMARK checklist^1^.**

| **Item to be reported** | | **Page no.** |
| --- | --- | --- |
| **INTRODUCTION** | |  |
| 1 | State the marker examined, the study objectives, and any pre-specified hypotheses. | 3-4 |
| **MATERIALS AND METHODS** | |  |
| *Patients* | |  |
| 2 | Describe the characteristics (e.g., disease stage or co-morbidities) of the study patients, including their source and inclusion and exclusion criteria. | 5, Table 1, S. figure 1 |
| 3 | Describe treatments received and how chosen (e.g., randomized or rule-based). | 5 |
| *Specimen characteristics* | |  |
| 4 | Describe type of biological material used (including control samples) and methods of preservation and storage. | 5 |
| *Assay methods* | |  |
| 5 | Specify the assay method used and provide (or reference) a detailed protocol, including specific reagents or kits used, quality control procedures, reproducibility assessments, quantitation methods, and scoring and reporting protocols. Specify whether and how assays were performed blinded to the study endpoint. | 5-12, S. tables 2 and 3 |
| *Study design* | |  |
| 6 | State the method of case selection, including whether prospective or retrospective and whether stratification or matching (e.g., by stage of disease or age) was used. Specify the time period from which cases were taken, the end of the follow-up period, and the median follow-up time. | 5 |
| 7 | Precisely define all clinical endpoints examined. | 11 |
| 8 | List all candidate variables initially examined or considered for inclusion in models. | 11-12 |
| 9 | Give rationale for sample size; if the study was designed to detect a specified effect size, give the target power and effect size. | 5 |
| *Statistical analysis methods* | |  |
| 10 | Specify all statistical methods, including details of any variable selection procedures and other model-building issues, how model assumptions were verified, and how missing data were handled. | 11-12 |
| 11 | Clarify how marker values were handled in the analyses; if relevant, describe methods used for cutpoint determination. | 8 |
| **RESULTS** | |  |
| *Data* | |  |
| 12 | Describe the flow of patients through the study, including the number of patients included in each stage of the analysis (a diagram may be helpful) and reasons for dropout. Specifically, both overall and for each subgroup extensively examined report the numbers of patients and the number of events. | S. figure 1 and in tables |
| 13 | Report distributions of basic demographic characteristics (at least age and sex), standard (disease-specific) prognostic variables, and tumor marker, including numbers of missing values. | Table 1, S.figure 1 |
| *Analysis and presentation* | |  |
| 14 | Show the relation of the marker to standard prognostic variables. | Tables 2 and 3 and S. tables |
| 15 | Present univariable analyses showing the relation between the marker and outcome, with the estimated effect (e.g., hazard ratio and survival probability). Preferably provide similar analyses for all other variables being analyzed. For the effect of a tumor marker on a time-to-event outcome, a Kaplan-Meier plot is recommended. | Figures 1-3, table 2 and S. tables and figures |
| 16 | For key multivariable analyses, report estimated effects (e.g., hazard ratio) with confidence intervals for the marker and, at least for the final model, all other variables in the model. | Tables 2-3, S. tables |
| 17 | Among reported results, provide estimated effects with confidence intervals from an analysis in which the marker and standard prognostic variables are included, regardless of their statistical significance. | Tables 2-3, S. tables |
| 18 | If done, report results of further investigations, such as checking assumptions, sensitivity analyses, and internal validation. | Reported in each table, if relevant |
| **DISCUSSION** | |  |
| 19 | Interpret the results in the context of the pre-specified hypotheses and other relevant studies; include a discussion of limitations of the study. | 18-20 |
| 20 | Discuss implications for future research and clinical value. | 18-20 |

Supplementary table 2**: Antibodies and reagents.**

| **Reagent** | **Dilution used** | **Incubation time (min)** | **Vendor** | **Product #** | **Used in stain number** |
| --- | --- | --- | --- | --- | --- |
| Anti-CD56 (Clone MRQ-42) | 1:400 | 30 | Cell Marque | 156R-96 | 1 |
| Anti-CD8 (Clone C8/144B) | 1:800 | 30 | DAKO/Agilent | M710301-2 | 1 & 2 |
| Anti-CD3 (Clone F7.2.38) | 1:300 | 30 | DAKO/Agilent | M7254 | 1 |
| Anti-E-cadherin (Clone 36) | 1:20000 | 30 | BD-Biosciences | 610182 | 1 |
| Anti-Cytokeratin (C-11) | 1:4000 | 30 | Abcam | 7753 | 1 |
| Anti-Cytokeratin (AE1/AE3, Type I/II) | 1:2000 | 30 | Life Technologies/Thermo Fisher Scientific | MA5 - 13156 | 1 |
| Anti-CD4 (Clone EP204) | 1:50 | 30 | Cell Marque | 104R-26 | 2 |
| Anti-FOXP3 (Clone D6O8R) | 1:100 | 30 | Cell Signaling Technology | 12653 | 2 |
| Anti-CD25 (Clone EP218) | 1:100 | 30 | Cell Marque | AC-0209 | 2 |
| 3-in-1 high-pH buffer | 1:50 | 20 | DAKO/Agilent | K8004 | 1 & 2 |
| 3-in-1 low-pH buffer | 1:50 | 20 | DAKO/Agilent | K8005 | 1 |
| High-pH buffer | 1:10 | 20 | PerkinElmer/Akoya | AR9001KT | 2 |
| Low-pH buffer | 1:10 | 20 | PerkinElmer/Akoya | AR6001KT | 2 |
| Blocking buffer/primary antibody dilution buffer | - | 10 | PerkinElmer/Akoya | In 4-plex kit NEL810001KT | 1 & 2 |
| Secondary antibody (anti-rabbit/anti-mouse) | - | 10 | PerkinElmer/Akoya | In 4-plex kit NEL810001KT | 1 & 2 |
| Opal fluorophores | Varies, see supplementary table 3 | 10 | PerkinElmer/Akoya | In 4-plex kit NEL810001KT and FP1495001KT | 1 & 2 |
| DAPI | 1 drop per mL | 5 | PerkinElmer/Akoya | In 4-plex kit NEL810001KT | 1 & 2 |
| Prolong Diamond Antifade Mountant | - | - | Life Technologies/ThermoFisher Scientific | P36970 | 1 & 2 |
| Wash buffer | 1:20 | 3 x 2 min between each step | DAKO/Agilent | K8007 | 1 & 2 |

Supplementary table 3**: Brief overview of staining sequence and fluorescent probe-pairing.** Antibody clones and dilutions are listed in supplementary table 2.

|  | **Deparaffinization/**  **Antigen retrieval** | **Ab 1 / Fluor** | **Ab-removal** | **Ab 2 / Fluor** | **Ab-removal** | **Ab 3 / Fluor** | **Ab-removal** | **Ab 4 / Fluor** | **Ab-removal** |
| --- | --- | --- | --- | --- | --- | --- | --- | --- | --- |
| Stain 1 (Pan/cytotoxic T cell stain) | 3-in-1 high pH buffer (DAKO) | Anti-CD56, Opal 620 (1:200) | 3-in-1 high pH buffer (DAKO) | Anti-CD8, Opal 520 (1:100) | 3-in-1 high pH buffer (DAKO) | Anti-CD3, Opal 570 (1:100) | 3-in-1 low pH buffer (DAKO) | Anti-E-cadherin/anti-cytokeratin (C-11)/anti-cytokeratin (Type I/II),  Opal 690 (1:100) | 3-in-1 low pH buffer (DAKO) |
| Stain 2 (Treg stain) | 3-in-1 high pH buffer (DAKO) | Anti-CD4, Opal 520 (1:100) | Low pH buffer (Akoya) | Anti-FOXP3, Opal 690 (1:100) | High pH buffer (Akoya) | Anti-CD25, Opal 620 (1:100) | High pH buffer (Akoya) | Anti-CD8,  Opal 570 (1:200) | Low pH buffer (Akoya) |

Abbreviations: Ab; antibody, Fluor; fluorescent probe.

Supplementary table 4**: Statistical analyses of included vs. excluded stage I-III R0 CRC patients.** Characteristics of included patients were compared to patients excluded due to technical reasons. Wilcoxon rank-sum test was used to test for association with age, Fisher Exact test for sex, MSI-, *BRAF*^V600E^- and *KRAS* status, Chi-squared test for pT, pN and tumor location and log-rank test for five-year RFS.

|  | Levels | Included | Excluded | p-value |
| --- | --- | --- | --- | --- |
| Total patients |  | 1088 (85%) | 186 (15%) |  |
| Age | median (range) | 74 (27 – 97) | 71 (34 – 93) | 0.09 |
| Sex | Female | 579 (86%) | 96 (14%) | 0.69 |
|  | Male | 509 (85%) | 90 (15%) |  |
| pT | 1 | 60 (82%) | 13 (18%) | 0.30 |
|  | 2 | 221 (89%) | 28 (11%) |  |
|  | 3 | 754 (85%) | 137 (15%) |  |
|  | 4 | 53 (88%) | 7 (12%) |  |
|  | NA | 0 | 1 |  |
| pN | 0 | 741 (85%) | 133 (15%) | 0.67 |
|  | 1 | 245 (87%) | 37 (13%) |  |
|  | 2 | 99 (86%) | 16 (14%) |  |
|  | NA | 3 | 0 |  |
| Tumor location | Right colon | 474 (87%) | 71 (13%) | 0.31 |
|  | Left colon | 351 (85%) | 62 (15%) |  |
|  | Rectum | 263 (83%) | 53 (17%) |  |
| MSI status | MSI | 186 (89%) | 23 (11%) | 0.43 |
|  | MSS | 845 (87%) | 131 (13%) |  |
|  | NA | 57 | 32 |  |
| *BRAF*^V600E^ status | Wild-type | 859 (86%) | 141 (14%) | 0.019 |
|  | Mutated | 174 (92%) | 15 (8%) |  |
|  | NA | 55 | 30 |  |
| *KRAS* status | Wild-type | 453 (86%) | 73 (14%) | 1 |
|  | Mutated | 191 (86%) | 31 (14%) |  |
|  | NA | 444 | 82 |  |
| Five-year-RFS | Percent | 60 % | 63 % | 0.65 |

Abbreviations: MSI; microsatellite instable, MSS; microsatellite stable, NA; Not available, RFS; relapse-free survival.

Supplementary table 5**: Akaike’s information criterion for combinations of log2-transformed continuous CD3/CD8 scores in Cox-proportional hazards models, stratified by cohort.** Five-year RFS was used as endpoint in stage I-III patients with residual tumor status 0 (n = 1088, events = 434).

| **Variable(s) in model** | **AIC** | **HR (95% CI)** | **p-value** |
| --- | --- | --- | --- |
| ieCD3* | 5229 | 0.90 (0.87 – 0.93) | <0.0001 |
| ieCD8* | 5237 | 0.92 (0.89 – 0.94) | <0.0001 |
| sCD3 | 5228 | 0.87 (0.83 – 0.90) | <0.0001 |
| sCD8 | 5253 | 0.94 (0.91 – 0.97) | <0.0001 |
| ieCD3* ieCD8 | 5228 | - | - |
| ieCD8 sCD3 | 5221 | 0.95 (0.92 – 0.98) 0.90 (0.85 – 0.94) | 0.003 <0.0001 |
| ieCD3* sCD8 | 5230 | - | - |
| sCD3 sCD8 | 5230 | - | - |

All variables have been log2-transformed and are modeled as continuous linear variables. Abbreviations: ieCD3; intraepithelial CD3, ieCD8; intraepithelial CD8, sCD3; stromal CD3, sCD8; stromal CD8. * Violates proportional hazards assumption.

Supplementary table 6**: Akaike’s information criterion for combinations of median-dichotomized CD3/CD8 scores in Cox-proportional hazards models, stratified by cohort.** Five-year RFS was used as endpoint in stage I-III patients with residual tumor status 0 (n = 1088, events = 434).

| **Variable(s) in model** | **AIC** | **HR (95% CI)** | **p-value** |
| --- | --- | --- | --- |
| ieCD3 | 5236 | 0.57 (0.47 – 0.69) | <0.0001 |
| ieCD8* | 5233 | 0.56 (0.46 – 0.67) | <0.0001 |
| sCD3* | 5243 | 0.61 (0.50 – 0.74) | <0.0001 |
| sCD8 | 5250 | 0.65 (0.54 – 0.79) | <0.0001 |
| ieCD3  ieCD8 | 5226 | - | - |
| ieCD8*  sCD3 | 5225 | 0.63 (0.51 – 0.77)  0.72 (0.59 – 0.89) | <0.0001  0.002 |
| ieCD3  sCD8 | 5234 | - | - |
| sCD3*  sCD8 | 5242 | - | - |

All variables have been dichotomized at the median. Abbreviations: ieCD3; intraepithelial CD3, ieCD8; intraepithelial CD8, sCD3; stromal CD3, sCD8; stromal CD8. * Violates proportional hazards assumption.

Supplementary table 7**: Associations between intraepithelial CD8- and stromal CD3-infiltration and clinicopathological and molecular characteristics in stage I-III R0 CRC patients of the pooled series.** Intraepithelial CD8 and stromal CD3 were analyzed as log2-transformed continuous variables. Pearson correlation was used to test for associations with age, Wilcoxon rank-sum test for sex, MSI-, *BRAF*^V600E^- and *KRAS* status and Kruskal-Wallis for pT, pN and tumor location.

|  | Levels | n | ieCD8 Median (Q1-Q3) | p-value ieCD8 | sCD3 Median (Q1-Q3) | p-value sCD3 |
| --- | --- | --- | --- | --- | --- | --- |
| Total patients |  | 1088 | 4.9 (2.5 – 7.1) |  | 10.1 (8.7 – 11.1) |  |
| Age | median (range): 74 (27 – 97) | 1088 | 4.9 (2.5 – 7.1) | 0.088 | 10.1 (8.7 – 11.1) | 0.81 |
| Sex | Female | 579 | 5.1 (2.4 – 7.0) | 0.29 | 10.0 (8.6 – 11.1) | 0.10 |
|  | Male | 509 | 4.8 (2.5 – 7.1) |  | 10.3 (8.9 – 11.2) |  |
| pT | 1 | 60 | 5.8 (3.9 – 7.4) | <0.0001^+^ | 11.4 (10.2 – 12.0) | <0.0001^+^ |
|  | 2 | 221 | 5.7 (3.4 – 7.5) |  | 10.7 (9.6 – 11.5) |  |
|  | 3 | 754 | 4.6 (1.8 – 6.8) |  | 9.9 (8.5 – 11.0) |  |
|  | 4 | 53 | 4.5 (0 – 6.6) |  | 9.4 (8.3 – 10.3) |  |
| pN | 0 | 741 | 5.5 (3.0 – 7.5) | <0.0001^+^ | 10.3 (9.1 – 11.4) | <0.0001^+^ |
|  | 1 | 245 | 3.9 (0 – 5.8) |  | 9.5 (8.1 – 10.6) |  |
|  | 2 | 99 | 3.9 (0 – 6.3) |  | 9.7 (7.6 – 11.0) |  |
|  | NA | 3 | 3.9 (2.8 – 6.0) |  | 10.2 (9.5 – 10.3) |  |
| Tumor location | Right colon | 474 | 5.1 (2.6 – 7.7) | 0.002 | 10.1 (8.7 – 11.2) | 0.067^+^ |
|  | Left colon | 351 | 4.5 (1.8 – 6.4) |  | 10.0 (8.7 – 10.9) |  |
|  | Rectum | 263 | 5.1 (2.7 – 6.7) |  | 10.4 (8.8 – 11.3) |  |
| MSI status | MSI | 186 | 7.1 (4.2 – 8.8) | <0.0001 | 10.3 (9.1 – 11.3) | 0.13 |
|  | MSS | 845 | 4.6 (2.3 – 6.6) |  | 10.1 (8.7 – 11.1) |  |
|  | NA | 57 | 2.9 (0 – 6.2) |  | 9.6 (7.9 – 10.8) |  |
| *BRAF*^V600E^ status | Wild-type | 859 | 4.8 (2.4 – 6.7) | <0.0001 | 10.2 (8.9 – 11.2) | 0.27^+^ |
|  | Mutated | 174 | 6.0 (3.5 – 8.5) |  | 10.0 (8.5 – 11.2) |  |
|  | NA | 55 | 3.8 (0 – 6.8) |  | 9.4 (8.1 – 10.8) |  |
| *KRAS* status | Wild-type | 453 | 5.4 (2.6 – 7.6) | 0.098 | 10.0 (8.9 – 11.1) | 0.92 |
|  | Mutated | 191 | 4.6 (2.6 – 6.6) |  | 10.3 (8.6 – 11.2) |  |
|  | NA | 444 | 4.7 (1.9 – 6.7) |  | 10.2 (8.6 – 11.1) |  |

Abbreviations: ieCD8; intraepithelial CD8, MSI; microsatellite instable, MSS; microsatellite stable, NA; Not available, sCD3; stromal CD3.
^+^Significant when considering only MSS cancers; ieCD8 and sCD3 were inversely associated with pT and pN, sCD3 was higher in rectal cancers and sCD3 was lower in *BRAF*^V600E^ mutated tumors. No associations were significant when only considering MSI cancers.

Supplementary table 8**: Prognostic value of intraepithelial CD8- and stromal CD3-positive T-cell scores as log2-transformed continuous variables in stage I-III, R0 CRC patients.** Endpoint evaluated was five-year relapse-free survival. Analysis was stratified by cohort. Only patients with complete data for all variables were included in the analysis.

|  | Univariable analysis | | | Multivariable analysis  c-index (concordance): 0.687 (se = 0.013) | | |
| --- | --- | --- | --- | --- | --- | --- |
| Statistic | HR | 95% CI | p-value | HR | 95% CI | p-value |
| **ieCD8 (log2) *** | 0.91 | 0.89 – 0.94 | <0.0001 | 0.95 | 0.92 – 0.99 | 0.0078 |
| **sCD3 (log2)** | 0.87 | 0.83 – 0.91 | <0.0001 | 0.94 | 0.89 – 0.99 | 0.026 |
| **Sex** Women *vs* men | 0.99 | 0.81 – 1.20 | 0.89 | 0.84 | 0.69 – 1.03 | 0.097 |
| **Tumor Location**  Left *vs* right | 1.19 | 0.95 – 1.48 | 0.13 | 1.15 | 0.91 – 1.46 | 0.25 |
| Rectum *vs* right | 0.89 | 0.69 – 1.16 | 0.40 | 1.12 | 0.84 – 1.48 | 0.45 |
| **pT** T2 *vs* T1 | 0.99 | 0.56 – 1.76 | 0.98 | 0.89 | 0.50 – 1.59 | 0.70 |
| T3 *vs* T1 | 1.91 | 1.14 – 3.21 | 0.015 | 1.37 | 0.80 – 2.34 | 0.25 |
| T4 *vs* T1 | 3.30 | 1.78 – 6.11 | 0.00015 | 2.72 | 1.44 – 5.15 | 0.0021 |
| **pN** N1 vs N0 | 1.67 | 1.33 – 2.09 | <0.0001 | 1.51 | 1.19 – 1.91 | 0.00071 |
| N2 *vs* N0 | 2.66 | 1.99 – 3.56 | <0.0001 | 2.33 | 1.73 – 3.15 | <0.0001 |
| **MSI status** MSI *vs* MSS | 0.71 | 0.54 – 0.93 | 0.013 | 0.78 | 0.58 – 1.07 | 0.12 |
| **Age *** | 1.04 | 1.03 – 1.05 | <0.0001 | 1.04 | 1.03 – 1.05 | <0.0001 |

n = 1028, events = 406. Abbreviations: ieCD8; intraepithelial CD8, MSI; microsatellite instable, MSS; microsatellite stable, sCD3; stromal CD3.
*Violates proportional hazards assumption in univariable analysis.

Supplementary table 9**: Clinicopathological and immune cell density differences between stage I-III R0 and stage IV CRC patients.** Immune cell densities were analyzed as log2-transformed continuous variables. Wilcoxon rank-sum test was used to test for an association with age and the immune cell densities, Fisher Exact test for sex, MSI-, *BRAF*^V600E^- and *KRAS* status and Chi-squared test for tumor location.

|  |  | **Stage I-III, R0 patients** | **Stage IV patients** | **p-value** |
| --- | --- | --- | --- | --- |
| **Total patients, n** |  | 1088 | 228 |  |
| **Age** | Median (range) | 74 (27 – 97) | 69 (29 – 97) | <0.0001 |
| **Sex** | Female | 579 (53%) | 124 (54%) | 0.77 |
|  | Male | 509 (47%) | 104 (46%) |  |
| **Tumor location** | Right colon | 474 (44%) | 107 (47%) | 0.008 |
|  | Left colon | 351 (32%) | 87 (38%) |  |
|  | Rectum | 263 (24%) | 34 (15%) |  |
| **MSI status** | MSI | 186 (18%) | 16 (7%) | <0.0001 |
|  | MSS | 845 (82%) | 201 (93%) |  |
|  | NA | 57 | 11 |  |
| ***BRAF*^V6ooE^ mutational status** | Wild-type | 859 (83%) | 183 (84%) | 0.84 |
|  | Mutated | 174 (17%) | 35 (16%) |  |
|  | NA | 55 | 10 |  |
| ***KRAS* mutational status** | Wild-type | 453 (70%) | 84 (62%) | 0.07 |
|  | Mutated | 191 (30%) | 51 (38%) |  |
|  | NA | 444 | 93 |  |
| **Total CD3^+^-cells per mm^2^ (log2)** | Median (Q1-Q3) | 9.0 (7.4 – 10.1) | 8.1 (6.9 – 9.3) | <0.0001 |
| **Intraepithelial CD3^+^-cells per mm^2^ (log2)** | Median (Q1-Q3) | 6.8 (5.0 – 8.6) | 5.7 (3.5 – 7.6) | <0.0001 |
| **Stromal CD3^+^-cells per mm^2^ (log2)** | Median (Q1-Q3) | 10.1 (8.7 – 11.1) | 9.3 (8.0 – 10.4) | <0.0001 |
| **Total CD8^+^-cells per mm^2^ (log2)** | Median (Q1-Q3) | 6.2 (4.4 – 7.9) | 5.4 (3.4 – 7.1) | <0.0001 |
| **Intraepithelial CD8^+^-cells per mm^2^ (log2)** | Median (Q1-Q3) | 4.9 (2.5 – 7.1) | 3.1 (0 – 5.4) | <0.0001 |
| **Stromal CD8^+^-cells per mm^2^ (log2)** | Median (Q1-Q3) | 7.0 (5.2 – 8.6) | 6.5 (3.9 – 7.9) | 0.0004 |
| **Total FOXP3^+^-cells per mm^2^ (log2)** | Median (Q1-Q3) | 5.9 (4.3 – 7.4) | 5.4 (3.4 – 6.6) | <0.0001 |
| **Total tp-Treg-cells per mm^2^ (log2)** | Median (Q1-Q3) | 3.8 (1.8 – 5.2) | 3.3 (0.7 – 4.8) | 0.004 |

Abbreviations: MSI; microsatellite instable, MSS; microsatellite stable, NA; Not available, tp-Treg; triple-positive Treg.

Supplementary table 10**: Linear models for prediction of immune infiltration.** To determine whether there was a significant difference between stage I-III R0 and stage IV patients with respect to immune infiltration, variables with significant frequency differences between these two groups of patients were included in linear models for predicting densities of various immune cell populations.

|  | **Total CD3^+^-cells per mm^2^ (log2)** | **ieCD3^+^-cells per mm^2^ (log2)** | **sCD3^+^-cells per mm^2^ (log2)** | **Total CD8^+^-cells per mm^2^ (log2)** | **ieCD8^+^-cells per mm^2^ (log2)** | **sCD8^+^-cells per mm^2^ (log2)** | **Total Foxp3^+^-cells per mm^2^ (log2)** | **Total tp-Treg-cells per mm^2^ (log2)** |
| --- | --- | --- | --- | --- | --- | --- | --- | --- |
| Intercept | 9.1  (p < 0.0001) | 7.8  (p < 0.0001) | 9.9 (p < 0.0001) | 6.4  (p < 0.0001) | 5.5 (p < 0.0001) | 6.8 (p < 0.0001) | 5.9  (p < 0.0001) | 4.1  (p < 0.0001) |
| Age | 0.0009  (p = 0.84) | 0.001 (p = 0.84) | 0.002 (p = 0.69) | 0.009  (p = 0.12) | 0.01  (p = 0.09) | 0.01 (p = 0.13) | -0.002  (p = 0.70) | -0.005  (p = 0.30) |
| Location: Left *vs* right | -0.15  (p = 0.28) | -0.23 (p = 0.22) | -0.02 (p = 0.90) | -0.10  (p = 0.59) | -0.24  (p = 0.23) | -0.08 (p = 0.71) | 0.01  (p = 0.95) | -0.21  (p = 0.17) |
| Location: Rectum *vs* right | 0.15  (p = 0.36) | 0.11 (p = 0.62) | 0.25 (p = 0.11) | 0.09  (p = 0.66) | 0.16 (p = 0.49) | 0.17 (p = 0.46) | 0.34  (p = 0.07) | 0.22  (p = 0.23) |
| MSS *vs* MSI | -0.56  (p = 0.0008) | -1.56 (p < 0.0001) | -0.33 (p = 0.05) | -1.24  (p < 0.0001) | -1.93 (p < 0.0001) | -1.14 (p < 0.0001) | -0.27  (p = 0.16) | -0.14  (p = 0.46) |
| Stage IV *vs* Stage I-III | -0.73  (p < 0.0001) | -1.14 (p < 0.0001) | -0.73 (p < 0.0001) | -0.85  (p < 0.0001) | -1.26 (p < 0.0001) | -0.64 (p = 0.004) | -0.63  (p = 0.0004) | -0.44  (p = 0.01) |

Abbreviations: ieCD3; intraepithelial CD3, ieCD8; intraepithelial CD8, MSI; microsatellite instable, MSS; microsatellite stable, sCD3; stromal CD3, sCD8; stromal CD8, tp-Treg; triple-positive Treg.

Supplementary table 11**: Associations between spatial proximity of CD8+-cells and FOXP3+-cells or triple-positive Tregs and the intraepithelial CD8/stromal CD3 subgroups and MSI status in stage I-III R0 patients of the Norwegian series 2.**

|  | Spatial proximity test between CD8^+^- and FOXP3^+^-cells | | | Spatial proximity test between CD8^+^-cells and triple-positive Tregs | | |
| --- | --- | --- | --- | --- | --- | --- |
|  | Significant | Non-significant | p | Significant | Non-significant | p |
| Total | 207 (40%) | 310 (60%) |  | 115 (22%) | 402 (78%) |  |
|  |  |  |  |  |  |  |
| ieCD8^High^,sCD3^High^ | 79 (47%) | 90 (53%) | 0.015 | 51 (30%) | 118 (70%) | 0.009 |
| ieCD8^High^,sCD3^Low^ | 37 (44%) | 48 (56%) |  | 18 (21%) | 67 (79%) |  |
| ieCD8^Low^,sCD3^High^ | 38 (43%) | 51 (57%) |  | 20 (22%) | 69 (78%) |  |
| ieCD8^Low^,sCD3^Low^ | 53 (30%) | 121 (70%) |  | 26 (15%) | 148 (85%) |  |
|  |  |  |  |  |  |  |
| MSI | 39 (39%) | 60 (61%) | 0.91 | 19 (19%) | 80 (81%) | 0.50 |
| MSS | 165 (40%) | 246 (60%) |  | 94 (23%) | 317 (77%) |  |
| NA | 3 | 4 |  | 2 | 5 |  |

Supplementary table 12**: Associations between mean CD25 expression in triple-positive Tregs and clinicopathological and molecular characteristics in stage I-III R0 CRC patients of the pooled series.** Wilcoxon rank-sum test was used to test for an association with age and the immune cell densities, Fisher Exact test for sex, MSI-, *BRAF*^V600E^- and *KRAS* status and Chi-squared test for pT, pN and tumor location.

|  | Levels | Mean-CD25^High^ | Mean-CD25^Low^/tp-Treg^Negative^ | p-value |
| --- | --- | --- | --- | --- |
| Total patients |  | 157 (14%) | 931 (86%) |  |
| Age | median (range) | 72 (31 – 93) | 74 (27 – 97) | 0.21 |
| Sex | Female | 80 (14%) | 499 (86%) | 0.55 |
|  | Male | 77 (15%) | 432 (85%) |  |
| pT | 1 | 6 (10%) | 54 (90%) | 0.40 |
|  | 2 | 27 (12%) | 194 (88%) |  |
|  | 3 | 114 (15%) | 640 (85%) |  |
|  | 4 | 10 (19%) | 43 (81%) |  |
| pN | 0 | 90 (12%) | 651 (88%) | 0.005 |
|  | 1 | 46 (19%) | 199 (81%) |  |
|  | 2 | 21 (21%) | 78 (79%) |  |
|  | NA | 0 | 3 |  |
| Tumor location | Right colon | 66 (14%) | 408 (86%) | 0.89 |
|  | Left colon | 53 (15%) | 298 (85%) |  |
|  | Rectum | 38 (14%) | 225 (86%) |  |
| MSI status | MSI | 25 (13%) | 161 (87%) | 0.73 |
|  | MSS | 125 (15%) | 720 (85%) |  |
|  | NA | 7 | 50 |  |
| *BRAF*^V600E^ status | Wild-type | 127 (15%) | 732 (85%) | 0.64 |
|  | Mutated | 23 (13%) | 151 (87%) |  |
|  | NA | 7 | 48 |  |
| *KRAS* status | Wild-type | 62 (14%) | 391 (86%) | 0.18 |
|  | Mutated | 34 (18%) | 157 (82%) |  |
|  | NA | 61 | 383 |  |
| ieCD8 | median (Q1-Q3) | 3.2 (0 – 5.2) | 5.2 (2.8 – 7.3) | <0.0001 |
| sCD3 | median (Q1-Q3) | 9.0 (7.7 – 10.2) | 10.3 (9.0 – 11.3) | <0.0001 |

Abbreviations: ieCD8; intraepithelial CD8, MSI; microsatellite instable, MSS; microsatellite stable, NA; Not available, sCD3; stromal CD3, tp-Treg; triple-positive Treg.

Supplementary table 13**: Associations between CD8+-to-triple-positive Treg spatial groups and clinicopathological and molecular characteristics in stage I-III R0 CRC patients of the pooled series.** Wilcoxon rank-sum test was used to test for an association with age and the immune cell densities, Fisher Exact test for sex, mean CD25 expression, MSI-, *BRAF*^V600E^- and *KRAS* status and Chi-squared test for pT, pN and tumor location.

|  | Levels | Significant spatial proximity | Non-significant spatial proximity | p-value |
| --- | --- | --- | --- | --- |
| Total patients |  | 183 (18%) | 849 (82%) |  |
| Age | median (range) | 74 (32 – 97) | 74 (27 – 94) | 0.78 |
| Sex | Female | 88 (16%) | 463 (84%) | 0.12 |
|  | Male | 95 (20%) | 386 (80%) |  |
| pT | 1 | 11 (19%) | 48 (81%) | 0.96 |
|  | 2 | 37 (17%) | 179 (83%) |  |
|  | 3 | 127 (18%) | 578 (82%) |  |
|  | 4 | 8 (15%) | 44 (85%) |  |
| pN | 0 | 119 (17%) | 592 (83%) | 0.27 |
|  | 1 | 41 (18%) | 183 (82%) |  |
|  | 2 | 22 (23%) | 72 (77%) |  |
|  | NA | 1 | 2 |  |
| Tumor location | Right colon | 84 (18%) | 377 (82%) | 0.80 |
|  | Left colon | 54 (17%) | 272 (83%) |  |
|  | Rectum | 45 (18%) | 200 (82%) |  |
| MSI status | MSI | 32 (18%) | 150 (82%) | 0.92 |
|  | MSS | 145 (18%) | 653 (82%) |  |
|  | NA | 6 | 46 |  |
| *BRAF*^V600E^ status | Wild-type | 146 (18%) | 666 (82%) | 0.91 |
|  | Mutated | 31 (18%) | 138 (82%) |  |
|  | NA | 6 | 45 |  |
| *KRAS* status | Wild-type | 65 (15%) | 357 (85%) | 0.19 |
|  | Mutated | 36 (20%) | 146 (80%) |  |
|  | NA | 82 | 346 |  |
| ieCD8 | median (Q1-Q3) | 5.8 (4.0 – 7.5) | 4.9 (2.4 – 7.1) | 0.0004 |
| sCD3 | median (Q1-Q3) | 10.7 (9.8 – 11.8) | 10.1 (8.8 – 11.1) | <0.0001 |
| Mean CD25 expression in  tp-Tregs | Mean-CD25^High^ | 25 (17%) | 125 (83%) | 0.82 |
|  | Mean-CD25^Low^/tp-Treg^Negative^ | 158 (18%) | 724 (82%) |  |

Abbreviations: ieCD8; intraepithelial CD8, MSI; microsatellite instable, MSS; microsatellite stable, NA; Not available, sCD3; stromal CD3, tp-Treg; triple-positive Treg.

Supplementary table 14**: Results from bootstrap sampling and backward selection of variables.** Bootstrap sampling (x1000) with replacement was performed within the 950 patients with complete data for all specified variables. Each resulting sample was subjected to a backward selection procedure for inclusion in multivariable survival analysis. The number of times each variable was selected was recorded.

| **Variable** | **Hazard ratio in univariable survival analysis** | **p in univariable survival analysis** | **Included**  **(n times)** |
| --- | --- | --- | --- |
| **Age** | 1.03 | <0.0001 | 1000 |
| **pN:** pN1 *vs* pN0 | 1.75 | <0.0001 | 896 |
| **pN:** pN2 *vs* pN0 | 2.72 | <0.0001 | 998 |
| **Intraepithelial CD8 (continuous, log2-transformed)** | 0.91 | <0.0001 | 889 |
| **pT:** pT2 *vs* pT1 | 0.92 | 0.78 | 163 |
| **pT:** pT3 *vs* pT1 | 1.70 | 0.05 | 610 |
| **pT:** pT4 *vs* pT1 | 3.10 | 0.0004 | 968 |
| **CD8-tp-Treg spatial proximity:**  Significant *vs* non-significant | 1.33 | 0.03 | 644 |
| **MSI status:** MSI *vs* MSS | 0.71 | 0.02 | 487 |
| **Stromal CD3 (continuous, log2-transformed)** | 0.87 | <0.0001 | 418 |
| **Mean CD25 expression in**  **tp-Tregs:**  mean-CD25^High^ *vs* mean-CD25^Low^/tp-Treg^Negative^ | 1.54 | 0.001 | 410 |
| ***BRAF*^V600E^ status:** mutated *vs* wild-type | 1.02 | 0.90 | 216 |
| **Sex:** Female *vs* male | 1.00 | 1.00 | 285 |
| **Location:** Left *vs* right colon | 1.17 | 0.18 | 286 |
| **Location:** Rectum *vs* right colon | 0.89 | 0.41 | 143 |
| **CD8-FOXP3 spatial proximity:**  Significant *vs* non-significant | 1.09 | 0.46 | 99 |

Patients with complete data for all variables were included in the analysis (N = 950). Abbreviations: MSI; Microsatellite instable, MSS; Microsatellite stable, tp-Treg; triple-positive Treg.

Supplementary table 15**: Multivariable survival analysis of the continuous log2-transformed intraepithelial CD8 variable and triple-positive Treg scores.** The analysis was stratified by cohort and performed within stage I-III, R0 CRC patients. Endpoint evaluated was five-year relapse-free survival. Only patients with complete data for all variables were included in the analysis.

|  | Multivariable analysis c-index (concordance): 0.693 (se = 0.013) | | |
| --- | --- | --- | --- |
| Statistic | HR | 95% CI | p-value |
| **ieCD8 (continuous, log2-transformed)** | 0.93 | 0.90 – 0.97 | 0.00011 |
| **Mean CD25 expression in tp-Tregs**  High *vs* Low/tp-Treg^Negative^ | 1.36 | 1.04 – 1.77 | 0.023 |
| **CD8-tp-Treg spatial proximity**  Significant *vs* non-significant | 1.38 | 1.07 – 1.77 | 0.014 |
| **pT**  T3 *vs* T1/2 | 1.51 | 1.15 – 2.00 | 0.0034 |
| T4 *vs* T1/2 | 2.97 | 1.92 – 4.61 | <0.0001 |
| **pN** N1 vs N0 | 1.49 | 1.16 – 1.90 | 0.0017 |
| N2 *vs* N0 | 2.27 | 1.67 – 3.10 | <0.0001 |
| **MSI status** MSI *vs* MSS | 0.73 | 0.55 – 0.98 | 0.036 |
| **Age** | 1.04 | 1.03 – 1.05 | <0.0001 |

n = 977, events = 376. Abbreviations: ieCD8; intraepithelial CD8, MSI; microsatellite instable, MSS; microsatellite stable, tp-Treg; triple-positive Treg.

Supplementary table 16**: Multivariable survival analysis of intraepithelial CD8 and tp-Treg scores, also including the stromal CD3 score.** The analysis was stratified by cohort and performed within stage I-III, R0 CRC patients. Endpoint evaluated was five-year relapse-free survival. Only patients with complete data for all variables were included in the analysis.

|  | Multivariable analysis c-index (concordance): 0.698 (se = 0.013) | | |
| --- | --- | --- | --- |
| Statistic | HR | 95% CI | p-value |
| **ieCD8**  High *vs* low | 0.65 | 0.52 – 0.81 | 0.0002 |
| **sCD3**  High *vs* low | 0.82 | 0.66 – 1.03 | 0.09 |
| **Mean CD25 expression in tp-Tregs**  High *vs* Low/tp-Treg^Negative^ | 1.30 | 0.99 – 1.70 | 0.05 |
| **CD8-tp-Treg spatial proximity**  Significant *vs* non-significant | 1.39 | 1.08 – 1.79 | 0.01 |
| **pT**  T3 *vs* T1/2 | 1.46 | 1.11 – 1.93 | 0.008 |
| T4 *vs* T1/2 | 2.82 | 1.81 – 4.39 | <0.0001 |
| **pN** N1 vs N0 | 1.50 | 1.17 – 1.92 | 0.001 |
| N2 *vs* N0 | 2.34 | 1.72 – 3.19 | <0.0001 |
| **MSI status** MSI *vs* MSS | 0.71 | 0.53 – 0.94 | 0.02 |
| **Age** | 1.04 | 1.03 – 1.05 | <0.0001 |

n = 977, events = 376. Abbreviations: ieCD8; intraepithelial CD8, MSI; microsatellite instable, MSS; microsatellite stable, sCD3; stromal CD3, tp-Treg; triple-positive Treg.

Supplementary table 17**: Multivariable survival analysis of intraepithelial CD8 and tp-Treg scores, including adjuvant chemotherapy as a covariable.** The analysis was stratified by cohort and performed within stage I-III, R0 CRC patients. Endpoint evaluated was five-year relapse-free survival. Only patients with complete data for all variables were included in the analysis. Of the 977 patients included in the model, 127 had received adjuvant chemotherapy while the remaining 850 did not.

|  | Multivariable analysis c-index (concordance): 0.695 (se = 0.013) | | |
| --- | --- | --- | --- |
| Statistic | HR | 95% CI | p-value |
| **ieCD8**  High *vs* low | 0.62 | 0.50 – 0.76 | <0.0001 |
| **Mean CD25 expression in tp-Tregs**  High *vs* Low/tp-Treg^Negative^ | 1.35 | 1.03 – 1.76 | 0.03 |
| **CD8-tp-Treg spatial proximity**  Significant *vs* non-significant | 1.36 | 1.06 – 1.75 | 0.02 |
| **pT**  T3 *vs* T1/2 | 1.50 | 1.14 – 1.98 | 0.004 |
| T4 *vs* T1/2 | 2.98 | 1.92 – 4.62 | <0.0001 |
| **pN** N1 vs N0 | 1.52 | 1.17 – 1.97 | 0.001 |
| N2 *vs* N0 | 2.31 | 1.67 – 3.19 | <0.0001 |
| **MSI status** MSI *vs* MSS | 0.72 | 0.54 – 0.96 | 0.03 |
| **Age** | 1.04 | 1.03 – 1.05 | <0.0001 |
| **Adjuvant chemotherapy**  Yes *vs* No | 1.05 | 0.74 – 1.50 | 0.78 |

n = 977, events = 376. Abbreviations: ieCD8; intraepithelial CD8, MSI; microsatellite instable, MSS; microsatellite stable, sCD3; stromal CD3, tp-Treg; triple-positive Treg.

Supplementary table 18**: Final model evaluated in microsatellite instable patients only.**

|  | Multivariable analysis in MSI patients c-index (concordance): 0.748 (se = 0.033) | | |
| --- | --- | --- | --- |
| Statistic | HR | 95% CI | p-value |
| **ieCD8**  High *vs* low | 0.54 | 0.29 – 0.99 | 0.048 |
| **Mean CD25 expression in tp-Tregs**  High *vs* Low/tp-Treg^Negative^ | 1.05 | 0.46 – 2.41 | 0.91 |
| **CD8-tp-Treg spatial proximity**  Significant *vs* non-significant | 2.73 | 1.45 – 5.13 | 0.0018 |
| **pT**  T3 *vs* T1/2 | 1.63 | 0.74 – 3.58 | 0.22 |
| T4 *vs* T1/2 | 6.34 | 2.14 – 18.79 | 0.00086 |
| **pN** N1 vs N0 | 2.56 | 1.36 – 4.83 | 0.0038 |
| N2 *vs* N0 | 2.29 | 0.96 – 5.45 | 0.063 |
| **Age** | 1.07 | 1.03 – 1.10 | <0.0001 |

n = 182, events = 57. Abbreviations: ieCD8; intraepithelial CD8, tp-Treg; triple-positive Treg.

Supplementary table 19**: Final model evaluated in microsatellite stable patients only.** The model violated the proportional hazards assumption. The model was therefore also evaluated with the continuous log2-transformed ieCD8 variable; this model did not violate the proportional hazards assumption and provided similar results (data not included).

|  | Multivariable analysis in MSS patients c-index (concordance): 0.687 (se = 0.014) | | |
| --- | --- | --- | --- |
| Statistic | HR | 95% CI | p-value |
| **ieCD8**  High *vs* low | 0.60 | 0.48 – 0.76 | <0.0001 |
| **Mean CD25 expression in tp-Tregs**  High *vs* Low/tp-Treg^Negative^ | 1.41 | 1.06 – 1.87 | 0.017 |
| **CD8-tp-Treg spatial proximity**  Significant *vs* non-significant | 1.25 | 0.95 – 1.65 | 0.11 |
| **pT**  T3 *vs* T1/2 | 1.52 | 1.13 – 2.05 | 0.0058 |
| T4 *vs* T1/2 | 2.68 | 1.64 – 4.39 | <0.0001 |
| **pN** N1 vs N0 | 1.44 | 1.11 – 1.87 | 0.0068 |
| N2 *vs* N0 | 2.33 | 1.67 – 3.26 | <0.0001 |
| **Age** | 1.04 | 1.03 – 1.05 | <0.0001 |

n = 795, events = 319. Abbreviations: ieCD8; intraepithelial CD8, tp-Treg; triple-positive Treg.

## Supplementary reference

1. McShane LM, Altman DG, Sauerbrei W, Taube SE, Gion M, Clark GM, et al. Reporting recommendations for tumor marker prognostic studies (REMARK). *J Natl Cancer Inst* **97**, 1180–4 (2005).
